# Distinctive Behavior and Selective Modulation of PPARγ by Pentacyclic Triterpenoid Pomolic Acid and Hederagenin from *Rosa canina*

**DOI:** 10.1021/acs.jafc.5c17657

**Published:** 2026-04-30

**Authors:** Mariano Nicola-Llorente, Francisco J. Hermoso-Pinilla, Daniel Torres-Oteros, F. Javier Luque, Silvia Canudas, Pedro F. Marrero, Diego Haro, Joana Relat

**Affiliations:** † Department of Nutrition, Food Sciences and Gastronomy, School of Pharmacy and Food Sciences, Food Torribera Campus, 16724 University of Barcelona, Santa Coloma de Gramenet 08921, Spain; ‡ Institute of Nutrition and Food Safety of the University of Barcelona (INSA-UB), Maria de Maeztu Unit of Excellence, Santa Coloma de Gramenet 08921, Spain; § Institute of Biomedicine of the University of Barcelona (IBUB), Barcelona 08028, Spain; ∥ Institute of Theoretical and Computational Chemistry (IQTCUB). Barcelona 08028, Spain; ⊥ Centro de Investigación Biomédica en Red de Fisiopatología de la Obesidad y Nutrición (CIBEROBN), Instituto de Salud Carlos III, Madrid 28029, Spain

**Keywords:** food bioactives, pentacyclic triterpenoids, *Rosa canina*, pomolic acid, hederagenin, TR-FRET binding assays, antagonists, selective PPARγ modulators, adipogenesis, lipid metabolism, functional foods, molecular docking and dynamics

## Abstract

Bioactive triterpenoids present in plant-derived foods are emerging as modulators of metabolic health, although their molecular targets and mechanisms remain unclear. In this study, we characterize Pomolic acid and Hederagenin, two pentacyclic triterpenoids from *Rosa canina*, as antagonists and selective modulators of peroxisome proliferator-activated receptor gamma (PPARγ). Both compounds reduced lipid accumulation during 3T3-L1 adipocyte differentiation and antagonized rosiglitazone-induced PPARγ transactivation without intrinsic agonist activity. Pomolic acid behaved as a neutral antagonist, repressing adipogenic and lipogenic gene expression and preventing TRAP220 recruitment. In contrast, Hederagenin selectively modulated PPARγ target genes involved in lipid handling while limiting triglyceride accumulation. TR-FRET assays confirmed direct binding to the receptor, and molecular dynamics simulations revealed a betulinic acid—like binding mode that destabilizes helices H11–H12 and disrupts the AF-2 coactivator interface. These findings provide mechanistic insight into how structurally related dietary triterpenoids modulate PPARγ signaling and support them as candidates for metabolic disease strategies.

## Graphical abstract

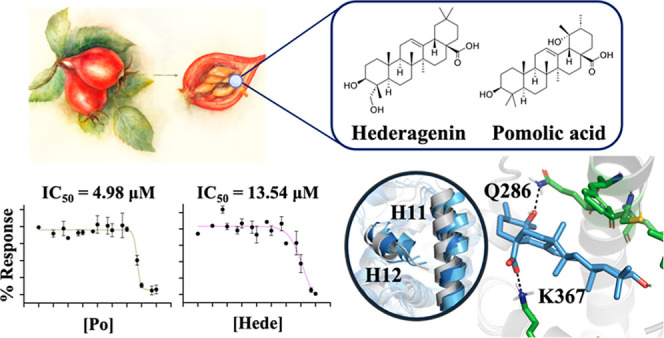

## Introduction

Obesity and its associated comorbidities, including type 2 diabetes, dyslipidemia, and metabolic dysfunction—associated steatotic liver disease, constitute a major and growing global health burden.^1^ Central to the pathophysiology of these disorders is the dysregulation of lipid storage and insulin sensitivity,^2^ which are processes tightly controlled by the nuclear receptor peroxisome proliferator-activated receptor gamma (PPARγ). PPARγ is a ligand-activated nuclear receptor that orchestrates adipocyte differentiation, lipid uptake and storage, and insulin sensitization, thereby exerting profound effects on whole-body metabolic homeostasis. Pharmacological activation of PPARγ with full agonists such as thiazolidinediones improves insulin sensitivity and glycemic control; however, their clinical utility is limited by adverse effects including weight gain, edema, cardiovascular risk, and bone loss.^3,4^ These drawbacks have fueled intense interest in alternative strategies aimed at selectively modulating PPARγ activity and avoid the receptor overactivation.^5,6^ In this context, partial agonists, antagonists, and so-called selective PPARγ modulators (SPPARγMs) have emerged as promising approaches to dissociate insulin-sensitizing actions from excessive adipogenesis and lipogenesis.^7–16^

Diet-derived bioactive compounds are increasingly recognized as relevant sources of PPARγ modulators with improved safety profiles.^17–19^ Among these, pentacyclic triterpenoids stand out due to their broad spectrum of biological activities, including anti-inflammatory, antiproliferative, and lipid-lowering effects.^20,21^ Several triterpenoids, such as betulinic acid and oleanolic acid derivatives, have been shown to antagonize or partially activate PPARγ, leading to reduced adiposity and improved metabolic outcomes in cell and preclinical models.^22–30^

*Rosa canina* (rosehip) is particularly rich in polyphenols and pentacyclic triterpenoids, and its consumption has been associated with beneficial metabolic effects.^31–43^ Previous work from our group demonstrated that dietary supplementation with *R. canina* flesh prevents high-fat-diet—induced obesity and improves glucose tolerance in mice, an effect linked to antagonism of PPARγ activity.^44^ Furthermore, the apolar (chloroform) fraction of *R. canina* pulp displayed the strongest biological activity in our previous experiments, significantly suppressing rosiglitazone-induced PPARγ activation in the HepG2 luciferase assay,^44^ and inhibiting 3T3-L1 adipocyte differentiation (unpublished data). The phytochemical profiling of this fraction revealed a high enrichment of the pentacyclic triterpenoids Pomolic acid (Po) and Hederagenin (Hede) (Figure 1).

**Figure 1:**
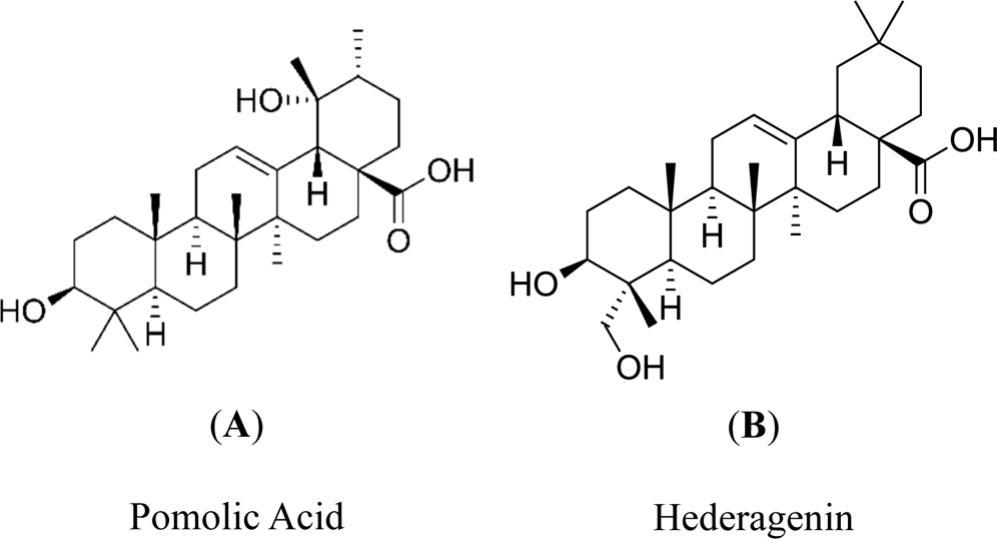
Chemical structures of Pomolic acid (Po) (A) and Hederagenin (Hede) (B). Structures highlight the shared pentacyclic triterpenoid scaffold and key functional groups.

Although other constituents of the fraction may contribute to its biological effects through potential synergistic interactions, the high abundance of these compounds suggested that they could be relevant contributors to the PPARγ-modulating activity of the extract. However, their specific role in mediating the observed bioactivity remained to be elucidated. Po and Hede have been reported to influence lipid metabolism and adipose tissue biology,^20,28,45^ yet their direct interaction with PPARγ and their functional impact on receptor signaling have not been comprehensively characterized. Notably, Po has been associated with reduced lipogenesis,^46,47^ whereas Hede has been linked to enhanced triglyceride clearance and adipose tissue browning,^28,48–50^ suggesting that closely related triterpenoids may exert distinctive metabolic effects.

Here, after isolation of Po and Hede from *R. canina* extracts, we report a detailed biochemical characterization of these compounds as natural PPARγ antagonists and dissect their distinct mechanisms of action using complementary cellular, biochemical, and computational approaches. By integrating adipocyte differentiation assays, transcriptional profiling, TR-FRET—based ligand and coregulator binding analyses, and molecular dynamics (MD) simulations, we provide mechanistic insight into how two structurally related triterpenoids can differentially modulate PPARγ activity. These findings advance our understanding of selective PPARγ modulation by dietary compounds and offer a molecular framework to explain the metabolic benefits associated with *R. canina* consumption.

## Materials and Methods

### Cell Culture

HepG2 (ATCC no. HB-8065) and 3T3-L1 (ATCC no. CL-173) cells were cultured in Dulbecco’s Modified Eagle’s Medium (DMEM) supplemented with 2 mM l-glutamine, 100 U/mL of penicillin G, 100 μg/mL streptomycin sulfate, and 10% fetal bovine serum (FBS) at 37 °C with 5% CO_2_ and saturated humidity (95%). Dimethyl sulfoxide (DMSO) was used as vehicle control at noncytotoxic concentrations (≤0.25%). All culture reagents were obtained from Thermo Fisher Scientific (USA).

### Membrane Integrity Analysis

Membrane integrity was assessed by dual staining with propidium iodide (PI; Sigma-Aldrich, USA) and Hoechst 33342 (Thermo Fisher Scientific, USA) after 24 h of treatment. HepG2 cells (5 × 10^5^ cells/mL) were seeded in 96-well plates 48 h prior to the treatments. Two control groups were included: solvent control (DMSO) and cytotoxic control (C400). The C400 control corresponds to the chloroform fraction of *R. canina* pulp at 400 μg/mL, a concentration previously established in our laboratory as cytotoxic in both PI and MTT assays that contains several of the bioactive molecules analyzed individually in this study. After 24 h, cells were washed with PBS and incubated for 15 min at 37 °C with the staining solution containing 10 μg/mL PI and 50 μM Hoechst in DMEM without phenol red. Hoechst staining allowed normalization of the PI fluorescence signal. After washing with PBS, 100 μL of phenol red-free DMEM was added, and fluorescence was measured using the Thermo Scientific Varioskan LUX multimode microplate reader (PI: ex535/em615 nm; Hoechst: ex369/em461 nm). Phenol red-free DMEM was used as the control medium for the readings.

### MTT Analysis

Cell viability was evaluated using the 3-(4,5-dimethylthiazol-2-yl)-2,5-diphenyltetrazolium bromide (MTT) assay (CyQUANT MTT Cell Proliferation Assay Kit, Invitrogen, USA). This assay is based on the ability of cells to reduce MTT to formazan. 3T3-L1 preadipocytes (2 × 10^4^ cells/mL) were seeded in 96-well plates 72 h before treatments. DMSO and ethanol (EtOH) served as solvent and cytotoxic controls, respectively. After 72 h of treatment, cells were washed with PBS and incubated for 3 h at 37 °C with 10 μL/well of 12 mM MTT and 100 μL of phenol red-free DMEM. Following incubation, 25 μL of the previous mixture was left in each well, and 50 μL of DMSO was added to dissolve the formazan. After 10 min at 37 °C, absorbance was measured at 540 nm using the Thermo Scientific Varioskan LUX multimode microplate reader.

### Adipocytes Differentiation

3T3-L1 preadipocytes (2 × 10^4^ cells/mL) were seeded in a 24-well plate on the day −3 (D-3). After 72 h (D0), the medium was replaced with Differentiation Medium (DMEM supplemented with the MDI cocktail including 1 μM dexamethasone, 500 μM 3-isobutyl-1-methylxanthine and 10 μg/mL insulin) to induce adipogenesis. Po and Hede were added at different concentrations through the differentiation of 3T3-L1 preadipocytes into mature adipocytes. From D3 to D6, the medium was replaced with DMEM supplemented with 10 μg/mL insulin. All chemical reagents were obtained from Sigma-Aldrich (USA). The MDI cocktail reagents were obtained from Sigma-Aldrich (USA), and Po and Hede were purchased from MedChemExpress (USA).

### Oil Red O Staining

Differentiated 3T3-L1 adipocytes were washed twice with PBS and fixed with 4% neutral-buffered formalin (Thermo Fisher Scientific, USA) for 30 min under gentle rotation. A 3 mg/mL Oil Red O (ORO, Sigma-Aldrich, USA) stock solution was prepared in isopropanol, and a working solution was obtained by mixing three parts of stock solution with two parts of water, followed by filtration through a 0.2 μm filter. After fixation, cells were rinsed twice with Milli-Q water, incubated with 60% isopropanol for 5 min, and then stained with the Oil Red O working solution for 15 min under gentle rotation. Following three washes with Milli-Q water and a 5 min-incubation with 60% isopropanol, cells were placed in water and photographed under an inverted microscope at 200× magnification. The dye was subsequently eluted with 100% isopropanol, and absorbance was measured at 490 nm using the Thermo Scientific Varioskan LUX multimode microplate reader. Isopropanol (100%) was used as a solvent control.

### Triglyceride Analysis Content

Cellular triglyceride (TAG) content was determined using the Triglyceride Quantification Colorimetric Kit (AssayGenie, Ireland), following the manufacturer’s instructions. 3T3-L1 cells were washed once with PBS, collected, and centrifuged at 1000*g* for 10 min. The pellet was resuspended in isopropanol, sonicated for 5 min and centrifuged again at 10,000*g* for 10 min. The supernatant was used for TAG analysis in 96-well plates alongside blanks (Milli-Q water) and standards (2.26 mM glycerol). Absorbance was measured at 510 nm using Thermo Scientific Varioskan LUX multimode microplate reader, and TAG concentrations were normalized to total protein content determined by the Bradford assay following the manufacturer’s instructions (Thermo Fisher Scientific, USA).

### Gene Expression Analysis

Total RNA from 3T3-L1 cells was extracted using TRI reagent solution (Thermo Fisher Scientific, USA) and treated with DNase I (Thermo Fisher Scientific, USA). cDNA was synthesized using the High-Capacity cDNA Reverse Transcription Kit (Thermo Fisher Scientific, USA) from 1.5 μg of total RNA. Relative mRNA expression was quantified by real-time quantitative PCR (RT-qPCR) using SBYR Master Mix CFX (Thermo Fisher Scientific, USA). Actin, M36B4 and B2M served as reference genes, and expression levels were normalized using Bestkeeper software. Results were obtained by the relative standard curve method and expressed as fold change versus control. Primer sequences are listed in Supporting Information Table S1.

### Transactivation Assay

HepG2 cells (5 × 10^5^ cells/mL) were seeded in 48-well plates and transfected using lipofectamine 2000 reagent (Invitrogen, USA) at a ratio of 1:2 (lipofectamine/DNA). Cells were cotransfected with the reporter plasmid 3 × PPRETKluc (2 ng/μL; kindly provided by Dr. Villarroya’s Lab) together with the expression vectors of PPARγ (pSV-SPORT-PPARγ) and RXRα (pCDM8-RXRα) (0.4 ng/μL each) and the internal control pRL-CMV (0.04 ng/μL; Promega, USA) as an internal transfection control. After 24 h, cells were treated with rosiglitazone (10 μM; Sigma-Aldrich, USA), Po, or Hede for 24 h. Luciferase activity was measured using the Dual-Luciferase Reporter Assay System (Sigma-Aldrich, USA), according to the manufacturer’s instructions, on a Berthold Detection System Sirius luminometer.

### TR-FRET PPARγ Competitive Binding Assay

A time-resolved fluorescence resonance energy transfer (TR-FRET) PPARγ competitive binding assay (Invitrogen, USA) was performed following the manufacturer’s protocol. Terbium-labeled anti-GST antibody was incubated with GST-PPARγ-LBD, and the complex was dispensed into 384-well plates (Thermo Fisher Scientific, USA) preloaded with test compounds (GW1929, Po, and Hede) and Fluormone Pan-PPAR Green tracer. After 4 h incubation in dark at room temperature, fluorescence was measured (λ_ex = 340 nm; λ_em = 495/520 nm) using a Tecan Infinite M Plex reader. The inhibition constant (*K*_i_) was calculated using the Cheng–Prusoff equation according to the manufacturer’s instructions.

### TR-FRET Coregulator Binding Assay

Ligand profiles (agonist, antagonist or inverse agonist) were determined using a TR-FRET coregulator binding assay (Invitrogen, USA) following the manufacturer’s protocol. The assay contained a Tb-anti-GST Ab, GST-PPARγ-LBD, and a fluorescein-labeled coregulator peptide (TRAP220/DRIP-2 or NCoR-1 ID2; amino acid sequence in Supporting Information Table S2). Terbium-labeled anti-GST antibody was incubated with fluorescein coregulator peptide, and this complex was dispensed into a 384-well plate (Thermo Fisher Scientific, USA) preloaded with the test compounds and GST-PPARγ-LBD. After 4 h incubation in dark at room temperature, fluorescence was measured (λ_ex_ = 340 nm; λ_em_ = 495/520 nm) using a Tecan Infinite M Plex reader.

### Statistical Analysis

All data are presented as mean ± standard error of the mean (SEM) from at least two independent experiments. Statistical analyses were conducted using GraphPad Prism, version 10.1.1 (GraphPad, San Diego, CA, USA). The specific statistical tests applied are indicated in the figure captions of the corresponding graphs. Two-tailed unpaired Student’s *t*-tests (with Welch’s correction when required) were applied for pairwise comparisons. For multiple comparisons, one-way ANOVA followed by Dunnett’s or Tukey’s post hoc tests was used. In TR-FRET binding assays, IC_50_ and EC_50_ values were calculated by fitting the data to sigmoidal dose–response curves (four-parameter logistic model) using nonlinear regression. Differences were considered statistically significant when *p* < 0.05.

### Molecular Dynamics Simulations

The crystallographic structures of the PPARγ ligand binding domain (LBD) from PDB entries 5F9B and 5LSG were used to explore the binding mode of Po and Hede, given their chemical similarity to the crystallographic ligands caulophyllogenin (CA, 5F9B; Figure 2) and betulinic acid (BA; 5LSG; Figure 2). Noteworthy, inspection of the two X-ray structures revealed that CA and BA adopt distinct arrangements in the binding pocket. Thus, whereas the carboxylate group of CA is buried in the interior of the cavity, the corresponding chemical moiety of BA is spatially pointing toward helix H12. Accordingly, MD simulations were used to examine the binding mode of Po and Hede considering the two potential arrangements of CA and BA, which were used as control.

**Figure 2:**
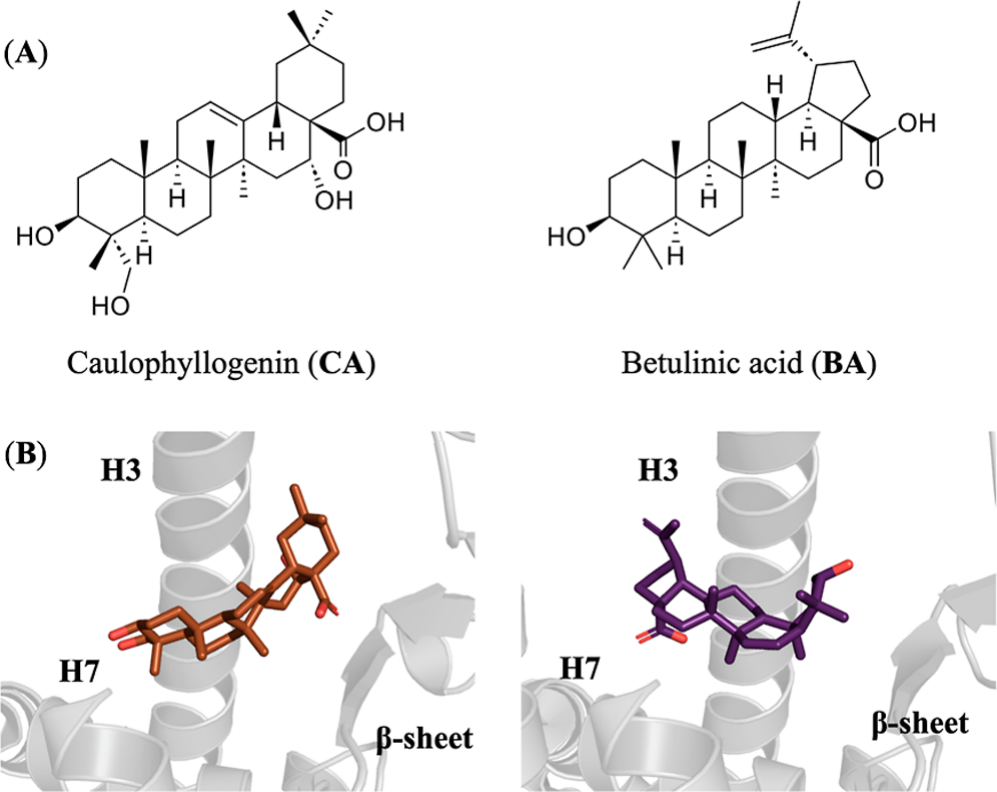
Schematic representation of caulophyllogenin and betulinic acid. (A) Chemical structures of the two compounds. (B) Representation of the binding mode to the LBD of PPARγ in the X-ray structures with PDB codes 5LSG (CA) and 5F9B (BA). One may notice the inverted arrangement of the ligands in the binding pocket.

The protein was modeled using the ff14SB force field.^51^ Due to the presence of gaps along the sequences in both structures (residues 259–274 and 264–275 in 5F9B and 5LSG, respectively), the final structures were built using homology modeling with SWISS-MODEL and the LBD protein sequence (UniProt code P37231).^52^ Since the X-ray structures were crystallized as dimers, only the ligand-bound chain (A in 5LSG and B in 5F9B) was selected for the simulations. H++ was employed to check the protonation state of protonatable residues.^53–56^ Considering the large structural similarity between the ligands, the binding mode of Po and Hede was obtained by overlapping their chemical structure onto the pose of CA and BA. All the ligands were parametrized using the general amber force field (GAFF2), and RESP charges were determined after full geometry optimization at the B3LYP/6-31G­(d) level^57–60^ using Gaussian.

For each PPARγ-ligand complex, the initial structures were solvated in a truncated octahedral box of 12 Å with ca. 13320 TIP3P water molecules.^61^ Neutralization of the system was achieved by adding K^+^ and Cl^–^ ions using the split-charge method in a 0.15 M concentration.^62^ The model systems were minimized in two steps (4000 energy minimization cycles with steepest descent, followed by 11,000 cycles of conjugate gradient minimization). Thermalization was accomplished in six steps by heating the systems from 100 to 300 K for a total of 300 ps (time step of 2 fs under the *NVT* conditions using the Langevin thermostat and a collision frequency of 1 ps^–1^). Next, the system was equilibrated at 300 K for 10 ns under *NPT* conditions with a constant pressure of 1 atm. Production simulations were carried out per triplicate with a simulation time of 1 μs per replica under the *NPT* ensemble. SHAKE was used in conjunction with a time step of 2 fs.^63^ Nonbonded interactions were cut at 10 Å, and the particle Mesh Ewald method was used for long-range electrostatic interactions.^64^ Temperature was kept at 300 K by the Berendsen thermostat. MD simulations were performed using the PMEMD.CUDA module of AMBER.

The binding free energy of the ligands was calculated using the molecular mechanics-generalized born surface area (MM-GBSA) methods. This analysis was performed for 200 snapshots taken regularly along the last 200 ns of the simulations. The binding free energy (Δ*G*_bind_; eq 1) was calculated as follows

1 $${\Delta G}_{bind}=G_{complex}-(G_{receptor}+G_{ligand})=\Delta H-T\Delta S$$

where *G*_complex_, *G*_receptor_ and *G*_ligand_ are the free energies of the complex, receptor and ligand, respectively.

The enthalpic component was determined from the difference between the MM energy (*E* ^MM^), and the solvation free energy (*G* ^solv^) of the bound (complex) and unbound (separate receptor and ligand) states (eq 2).

2 $$\Delta H={\Delta E}^{MM}{+\Delta G}^{solv}$$

The entropic contribution (*T*Δ*S*; eq 3) of the system was estimated following a statistical thermodynamics approach, calculated as follows

3 $$-T\Delta S=k_{B}T{Ln<e}_{\mathrm{int}}^{\beta \Delta E}>$$

where *k*_B_ is the Boltzmann’s constant, *T* is the temperature (298 K), β = 1/(*k*_B_ *T*), Δ*E*_int_ is the difference between the interaction energy of a given frame (*E*_int_) and the average value (⟨*E*_int_⟩) for the ensemble of frames, and the brackets stand for the average value of the exponential term.^65,66^ Calculations were performed using the MMPBSA.py module of AMBER employing the generalized born model (igb = 2) to estimate the solvation effects.

## Results

### Identification of the Nontoxic Concentrations of Pomolic Acid and Hederagenin in Hepatic and Adipocyte Cell Models

Dose–response analyses in HepG2 and 3T3-L1 cells established the concentration ranges at which Po and Hede did not induce cytotoxicity, as assessed by PI/Hoechst staining and MTT assays. In HepG2 cells, both membrane integrity (PI staining normalized to Hoechst) and mitochondrial function (MTT assay) remained unaffected after 24 h exposure to Po (0.5–5 μM) (Figure 3A,C) or Hede (1–25 μM) (Figure 3B,D). Although no significant cytotoxicity was detected, morphological alterations were observed at ≥5 μM Po (data not shown); therefore, concentrations above 1 μM were excluded from subsequent experiments in HepG2. In 3T3-L1 preadipocytes, MTT assays after 72 h revealed no cytotoxicity for Po (1.5–5 μM) or Hede (12–20 μM) (Figure 3E,F). PI staining proved unreliable in this cell line, but visual inspection and viability assays (MTT) confirmed the selected nontoxic ranges. Both compounds were well tolerated within the micromolar range used in subsequent experiments, consistent with previous reports identifying pentacyclic triterpenoids as low-toxicity natural metabolites.

**Figure 3:**
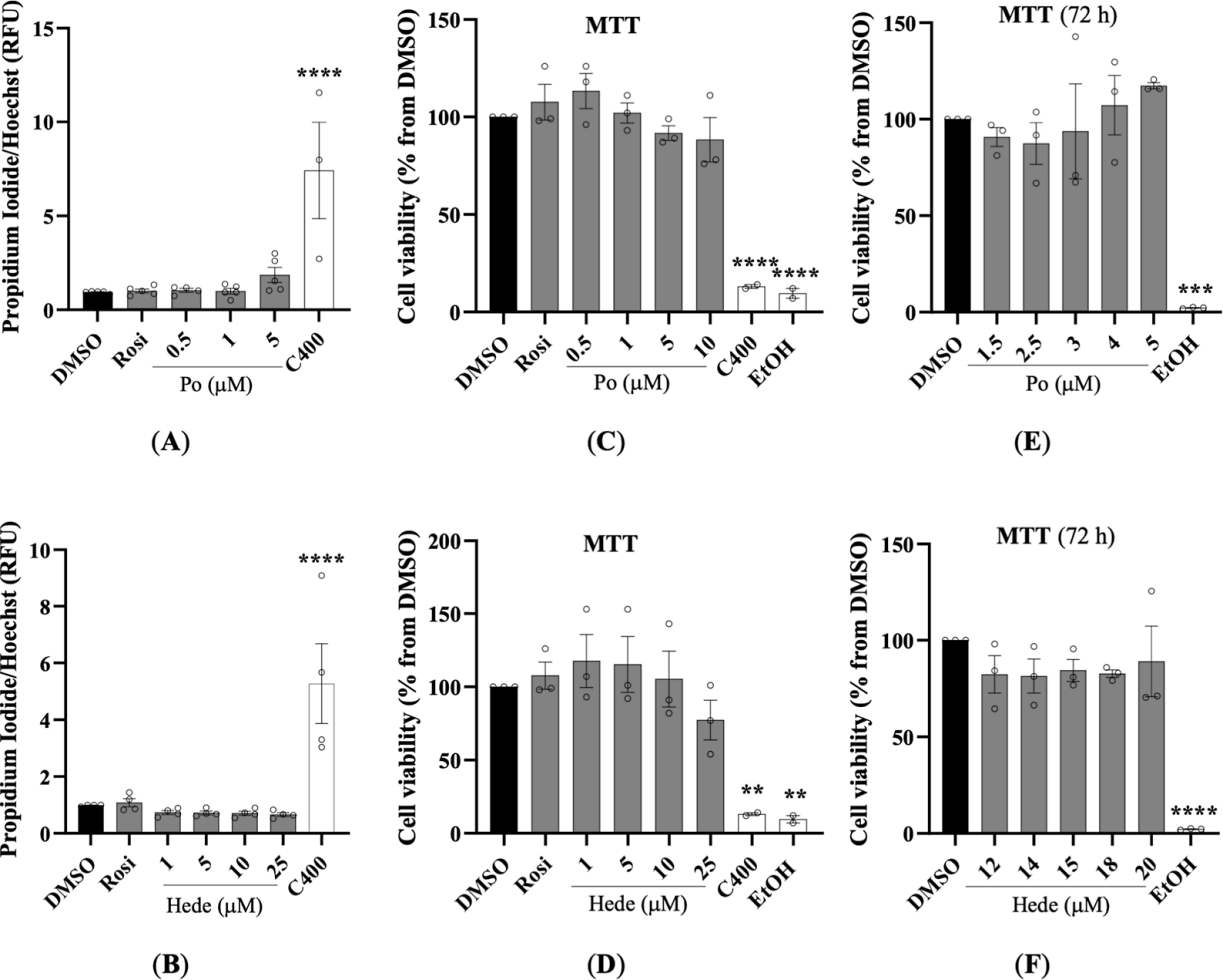
Effects of Pomolic acid and Hederagenin on cell viability. Effect of Po (0.5–5 μM) (A) or Hede (1–25 μM) (B) on membrane integrity in HepG2 cells, assessed by propidium iodide (PI) staining. HepG2 cells were treated with different concentrations of Po (*n* = 3–5), Hede (*n* = 3) or rosiglitazone (Rosi; 10 μM, included as control in each assay) for 24 h. Effect of Po, Hede or Rosi on mitochondrial activity in HepG2 (C,D) or 3T3-L1 cells (E,F) evaluated by MTT assay. Cells were treated for 24 h (HepG2) or 72 h (3T3-L1) with different concentrations as indicated. In cytotoxicity studies, C400 (internal cell death control) and/or 100% EtOH were used as positive controls. Data are presented as mean ± SEM. Statistical significance: *p < 0.05, **p < 0.01, ***p < 0.001, ****p < 0.0001 compared with control group (black bar in PI staining and MTT assay).

### Pomolic Acid and Hederagenin Antagonize PPARγ-Driven Adipogenesis through Distinct Transcriptional Programs

To investigate whether Po and Hede modulate PPARγ activity and considering the central role of this receptor in adipogenesis, their effects were evaluated in 3T3-L1 cells. 3T3-L1 preadipocytes were induced to differentiate into adipocytes using the standard MDI cocktail and treated with throughout both the induction (day 0) and maintenance (day 3–6) phases.

As shown in Figure 4, both compounds reduced intracellular lipid accumulation in differentiated 3T3-L1 cells, although their effects on the expression of individual PPARγ target genes differed. Treatment of differentiating 3T3-L1 adipocytes with Po (2.5–5 μM) or Hede (15–20 μM) markedly decreased lipid droplet formation as determined by Oil Red O staining (Figure 4A,D) and TAG quantification (Figure 4B,E).

**Figure 4:**
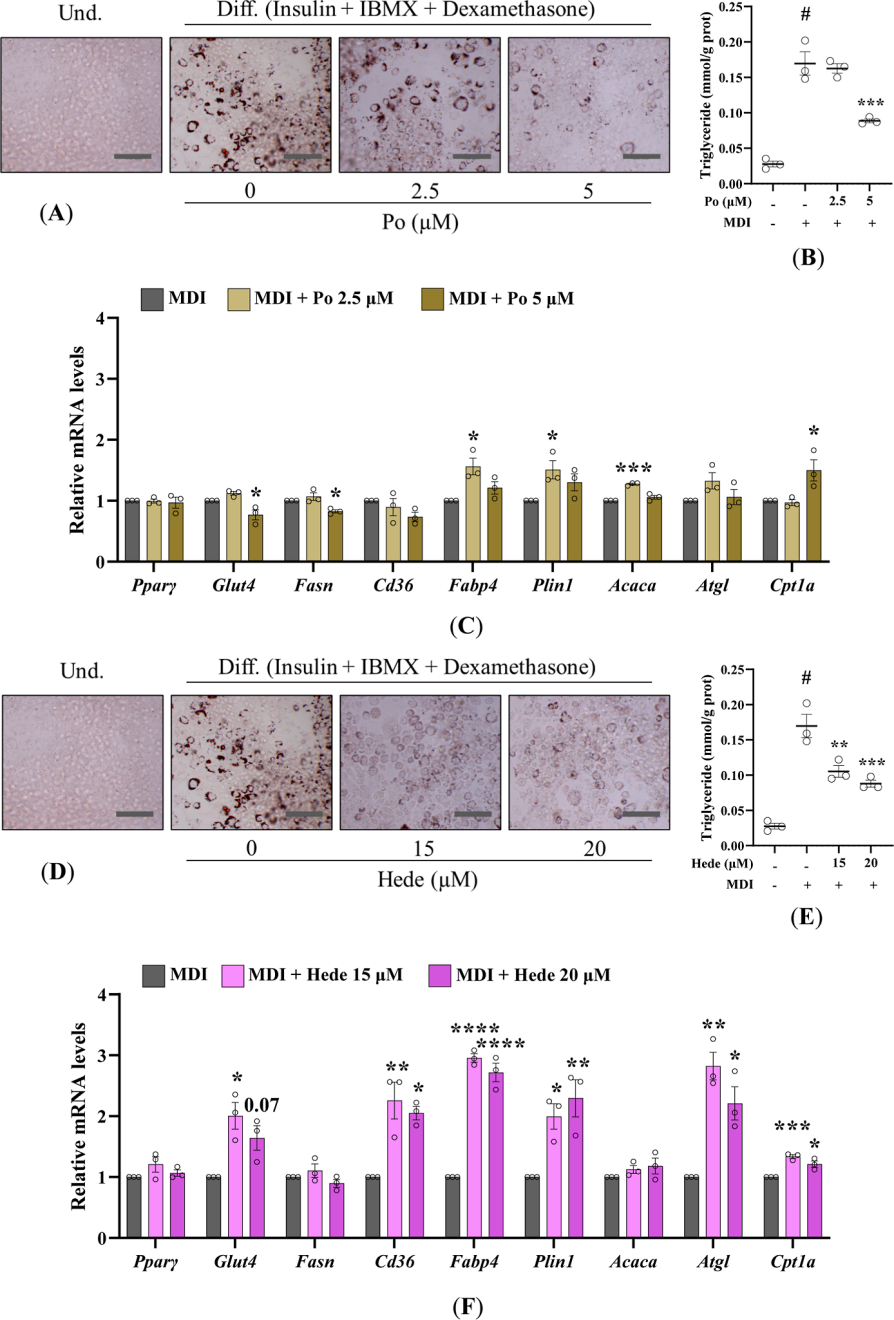
Pomolic acid and Hederagenin antagonize PPARγ-driven adipogenesis through distinct transcriptional programs. 3T3-L1 preadipocytes were differentiated using the MDI cocktail and treated with Po (2.5 and 5 μM) or Hede (15 and 20 μM). Representative ORO staining images (200× magnification; scale bar = 150 μm; *n* = 3) in Po (A) and Hede (D) treated cells. Total triglyceride quantification normalized to total protein (*n* = 3) in Po- (B) and Hede- (E) treated cells. Effects of Po- (C) and Hede- (F) on gene expression of adipogenic markers analyzed by RT-qPCR (*n* = 3). Data are presented as mean ± SEM. Statistical significance: *p < 0.05, **p < 0.01, ***p < 0.001, ****p < 0.0001, compared with untreated differentiated 3T3-L1 cells (MDI).

To gain mechanistic insight into the reduction of lipid accumulation observed under these conditions, the expression of genes involved in adipocyte lipid metabolism was analyzed to assess the transcriptional effects of Po and Hede. Basal expression in undifferentiated cells was included to illustrate gene induction during adipogenesis (Figure S1).

This analysis revealed distinct transcriptional responses. At 2.5 μM, Po elicited a moderate induction of several canonical PPARγ target genes such as *Fabp4*, *Plin1*, and *Acaca* yet this transcriptional activation did not translate into increased lipid accumulation (Figure 4C). When the concentration was increased to 5 μM, Po instead downregulated key lipogenic regulators, including *Glut4* and *Fasn* while upregulating *Cpt1a*, the rate-limiting enzyme in fatty acid oxidation, providing a mechanistic explanation for the reduced lipid droplet content observed under these conditions (Figure 4C). In contrast, Hede consistently enhanced the expression of PPARγ-dependent genes, notably *Glut4*, *Cd36*, *Fabp4*, and *Plin1* together with genes involved in lipid mobilization and fatty-acid oxidation, including *Atgl* and *Cpt1a* (Figure 4F). The analysis of all these genes provides a broader evaluation of the metabolic pathways affected by these compounds and supports a differential regulation of lipid metabolism by the two triterpenoids.

### Pomolic Acid and Hederagenin Antagonize Rosiglitazone-Induced PPARγ Transactivation

Luciferase reporter assays demonstrated that both Po and Hede dose-dependently antagonized rosiglitazone-induced transactivation of PPARγ in HepG2 cells. To assess this effect, HepG2 cells were cotransfected with a PPRE-driven luciferase reporter and expression vectors for PPARγ and RXRα. As expected, rosiglitazone (Rosi) markedly increased reporter activity, and this activation was progressively inhibited by Po (0.5–1 μM) and Hede (10–25 μM) (Figure 5A,B). Importantly, when the assay was performed in the absence of Rosi, neither compound displayed intrinsic agonist activity. Hede had no detectable effect on basal transcription, and Po produced only a slight reduction at 0.5 μM (Figure 5A,B).

**Figure 5:**
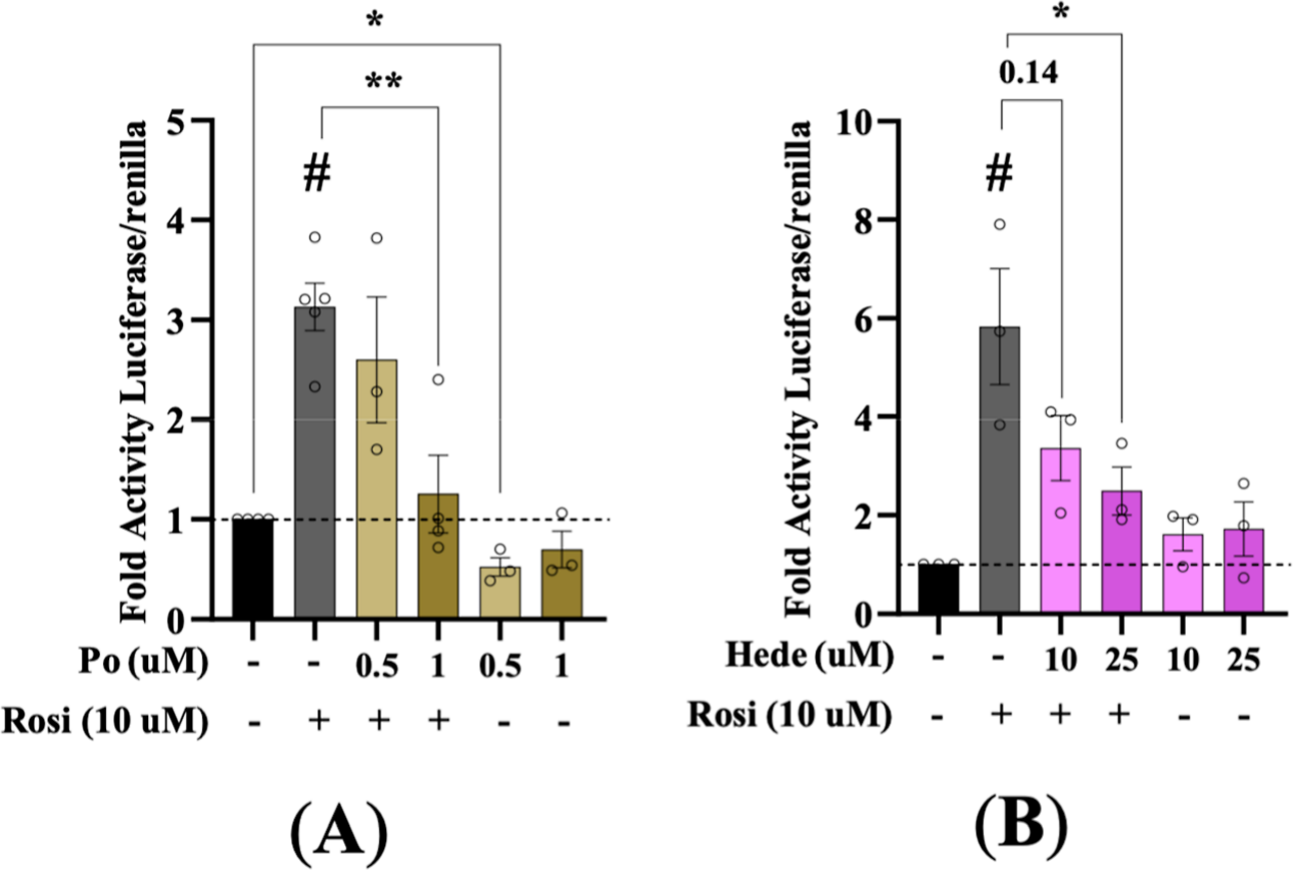
Pomolic acid and Hederagenin antagonize rosiglitazone-induced PPARγ transactivation. HepG2 cells were cotransfected with the 3xPPRE-luciferase reporter and expression plasmids for PPARγ and RXRα. After 24 h, cells were treated with either 10 μM of Rosi or DMSO, together with various concentrations of Po (0.5 and 1 μM) (A) or Hede (10 and 25 μM) (B) for an additional 24 h (*n* = 3–5). Data are presented as mean ± SEM. Statistical significance: *p < 0.05, **p < 0.01, compared with control group (positive control of rosiglitazone).

### Pomolic Acid and Hederagenin Directly Bind the PPARγ Ligand-Binding Domain and Differentially Modulate Coregulator Recruitment

PPARγ regulates gene transcription through dynamic and ligand-dependent interactions with coregulators, including coactivators that enhance transcription upon agonist binding (e.g., rosiglitazone and GW1929) and corepressors that maintain or reinforce transcriptional repression in the presence of antagonists or inverse agonists (e.g., GW9662 and T0070907). Full agonists and inverse agonists promote stable, well-defined receptor conformations that favor strong recruitment of coactivators or corepressors, respectively, resulting in divergent transcriptional outcomes.^67–69^ In contrast, antagonists and partial agonists stabilize a broader ensemble of receptor conformations, resulting in context-dependent patterns of coregulator engagement and transcriptional activity^67,69–71^ that vary with cell type, assay system, and coregulator availability.^11,26,69,72–75^ These regulatory differences ultimately arise from ligand-induced conformational rearrangements within the PPARγ ligand-binding domain (LBD), particularly affecting helix 12 positioning and the accessibility of coactivator and corepressor interaction surfaces.^69,76–78^

To evaluate whether the antagonist activity of Po and Hede arises from direct binding to the PPARγ LBD, a TR-FRET competitive ligand-binding assay was performed. The full agonist GW1929 (1 pM to 100 μM) was used as a positive control, and DMSO (1%) as a negative control. Both Po and Hede displaced the fluorescent ligand in a dose dependent manner over the tested concentration range (1 pM–100 μM). GW1929 induced a pronounced reduction in the TR-FRET ratio, exhibiting nanomolar potency (IC_50_ = 1.24 nM; *K*_i_ = 0.44 nM), in agreement with previous studies that reported similar inhibitory potency (*K*_i_ = 0.1 nM).^79^ In contrast, Po (IC_50_ = 4.98 μM; *K*_i_ = 1.79 μM; Figure 6A) and Hede (IC_50_ = 13.54 μM; *K*_i_ = 4.86 μM; Figure 6D) displayed moderate micromolar affinities, with Po being approximately 3-fold more potent. These results confirm that both compounds directly bind the PPARγ LBD, albeit with lower affinity than the full agonist.

**Figure 6:**
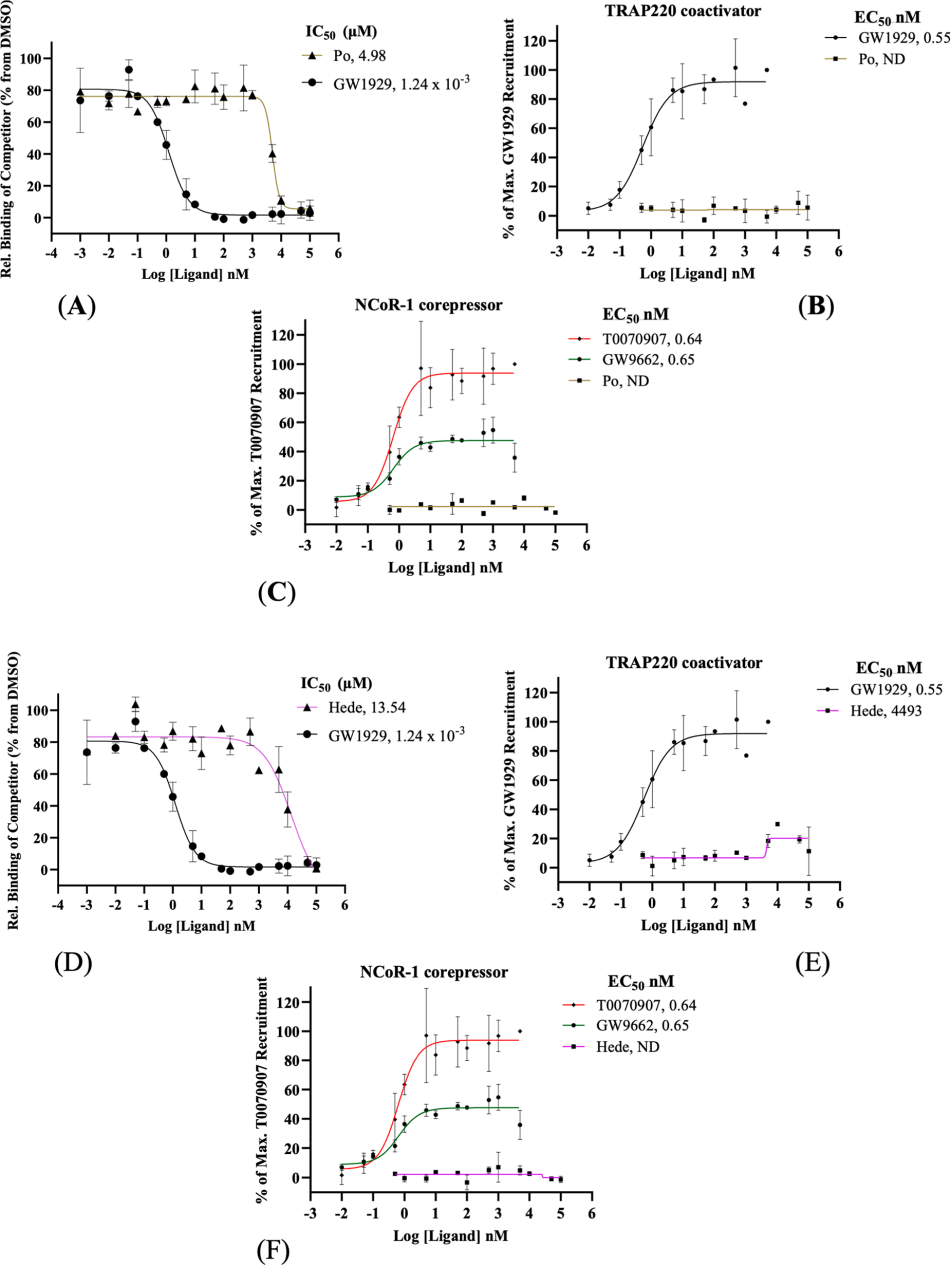
Pomolic acid and Hederagenin bind to the PPARγ LBD with distinct potencies and differential coregulator recruitment. TR-FRET competitive binding assays for GW1929 (positive control), Po (A), and Hede (D) were performed over a concentration range of 1 pM–100 μM (*n* = 2). TRAP220 coactivator recruitment assays are expressed as the % of the maximal response induced by GW1929 (positive control) for Po (B) and Hede (E) (1 pM–100 μM; *n* = 2). NCoR-1 corepressor recruitment assays expressed as the % of maximal response induced by T0070907 (positive control) for Po (C) and Hede (F) (1 pM–100 μM; *n* = 2). Dose–response curves were fitted using a sigmoidal model with variable slope in GraphPad Prism software. IC_50_ values were obtained from the curve fitting, and inhibition constants (*K*_i_) were calculated using the Cheng–Prusoff equation. Data are presented as mean ± SEM.

To further elucidate the molecular mechanism underlying PPARγ modulation, the ability of Po and Hede to recruit coactivators was assessed using a TR-FRET-based coregulator binding assays. As expected, GW1929 strongly promoted binding of the TRAP220 coactivator peptide (EC_50_ of 0.55 nM; maximal efficacy set at 100%). In contrast, Po did not induce detectable coactivator recruitment (Figure 6B), whereas Hede promoted a weak but still measurable TRAP220 interaction (EC_50_ = 4.49 μM), reaching approximately 19% of the maximal efficacy of GW1929 (Figure 6E).

To evaluate potential inverse agonist or antagonist activity, recruitment of the NCoR-1 corepressor was also examined. Neither Po nor Hede promoted NCoR-1 binding, in contrast to the inverse agonist T0070907 (EC_50_ = 0.64 nM, 100% efficacy) and the antagonist GW9662 (EC_50_ = 0.65 nM; ∼36% efficacy relative to T0070907) (Figure 6C,F). Taken together, these data indicate that Po functions as a silent or neutral antagonist, that binds the PPARγ LBD without promoting coactivator or corepressor recruitment. In contrast, Hede behaves as a noncanonical antagonist capable of eliciting partial coactivator engagement, consistent with stabilization of intermediate receptor conformations (Table 1).

**Table 1:** Binding Parameters

compound	IC_50_ (μM)	*K*_i_ (μM)	EC_50_ (μM), % TRAP220 recruitment
GW1929	0.0012	4.44 × 10^–4^	5.50 × 10^–4^, 100%
pomolic acid	4.98	1.79	ND, 0%
hederagenin	13.54	4.86	4.49, 19%

### Modeling the Binding of Pomolic Acid and Hederagenin to the LBD

In light of the previous results, we examined the putative binding mode of Po and Hede by using molecular modeling techniques. The structure of these compounds differs from the chemical scaffold of rosiglitazone, but it is similar to the structure of other pentacyclic triterpenoid compounds that are bound to the binding pocket of PPARγ, such as CA and BA. Thus, the structural similarity of Po and Hede determined using the Tanimoto index in conjunction with the molecular representation given by the Morgan fingerprint (radius 2) is less than 0.1 relative to rosiglitazone but ranges from 0.4 to 0.8 relative to CA and BA. Despite the presence of a common chemical scaffold in CA and BA, inspection of the X-ray structures show that these compounds adopt distinct arrangements in the binding pocket, as their orientations roughly reflect a 180-degree inversion of the chemical skeleton (Figure 2).

Given the distinct binding orientations of the control compounds CA and BA within the binding pocket of PPARγ, the interaction of Po and Hede with the PPARγ LBD was examined by modeling both compounds following the CA-like and BA-like arrangements. Three independent MD (1 μs) simulations were run per compound and binding mode to assess the structural and energetic features of ligand binding. Thus, a total of 24 MD simulations was performed for both control (CA, BA) and test (Po, Hede) compounds.

The root-mean square deviation (RMSD) profiles for the protein backbone, the subset of residues that shape the binding pocket, and ligand consistently supported the structural stability of both CA and BA in their respective native (crystallographic) complexes (Figure 7). Thus, the RMSD values determined for the protein backbone, binding site residues and ligand were generally close to 1.5–2.0 Å for CA in the CA-like binding mode, and similar trends were observed for BA in the BA-like pose (Figures S2 and S4). In contrast, the RMSD profiles revealed the occurrence of several structural fluctuations along the trajectories when CA was modeled in the BA-like binding mode and BA was modeled in the CA-like arrangement (Figures S2 and S4). In addition, larger root-mean square fluctuations (RMSF) were observed for the simulations run with CA in the BA-like arrangement and BA in the CA-like pose (Figures S3 and S5), especially regarding residues 225–275 and 350–370 and the C-terminus (helix 12), compared to the RMSF plots obtained for the native (X-ray) complexes of both CA and BA. This finding agrees with the larger structural changes observed in the RMSD plots. In addition, more compact structures are found for the complexes of CA in the CA-like binding pose, and BA in the BA-like arrangement, compared to the cross-bonded complexes (CA bound to 5LSG in the BA-like mode, and BA to 5F9B in the CA-like mode), as noted in the solvent-accessible surface area (SASA) of the ligand–protein complexes (see Supporting Information Table S3). Overall, these findings reflect the sensitivity of these structural analyses to support the arrangement of CA and BA in their respective crystallographic structures.

**Figure 7:**
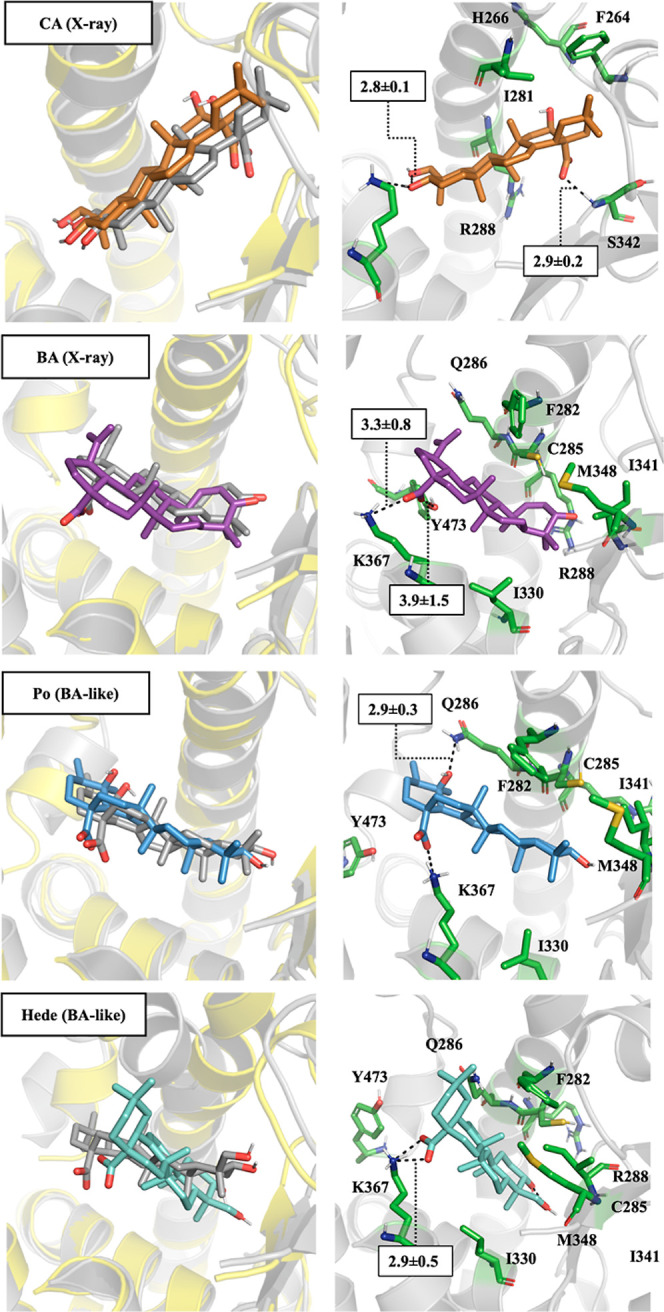
Representation of the most favorable binding mode obtained for CA, BA, Po and Hede from MD simulations. (Left) representation of the X-ray pose of the ligand (gray sticks; protein as yellow cartoon) and the final snapshot obtained at the end of the three MD simulations (CA: orange sticks, BA: magenta sticks; Po: blue sticks, Hede: pale green sticks; protein as gray cartoon). (Right) representation of selected interactions. Distances are the average value determined for the last 200 ns (Å; standard deviation in parentheses).

MM-GBSA calculations were used to estimate the binding affinity of CA and BA to their own X-ray structures (CA in 5F9B and BA in 5LSG) and in the complexes obtained upon cross-binding. Gratifyingly, the results supported the greater energetic stability of the native X-ray complexes formed with CA and BA (Table 2), particularly in this latter case. Despite the uncertainties of the MM-GBSA method, these results give confidence in the use of this computational approach to examine the binding mode of the structurally related Po and Hede to the PPARγ LBD.

**Table 2:** Binding-Free Energies of Each System from MM-GBSA (ε = 2) for the Last 200 ns of the Simulation

compound	*K*_d_^exp^ (μM)	binding mode	*E*_int_ (*E* ^ELE^, *E* ^vdW^)	Δ*G*_sol_	Δ*H*	*T*Δ*S*	Δ*G* ^MM/GBSA^
CA	54.82 ^80^	CA (X-ray 5F9B)	–50.3 (8.6, −58.9)	8.2	–42.1	–36.9	**–5.2** **±** **0.6**
		BA-like	–81.6 (−27.2, −54.4)	43.4	–38.2	–35.7	–2.5 ± 0.4
BA	4.00 ^23^	CA-like	–60.4 (−3.2, −57.3)	21.3	–39.1	–24.6	–14.5 ± 1.3
		BA (X-ray 5LSG)	–81.3 (−26.7, −54.6)	32.4	–48.9	–24.6	**–24.3** **±** **1.8**
PO	1.79	CA-like	–53.9 (2.0, −55.8)	14.1	–39.8	–33.6	–6.2 ± 3.2
		BA-like	–91.2 (−30.0, −61.2)	35.0	–56.2	–23.0	**–33.2** **±** **0.1**
HEDE	4.86	CA-like	–64.7 (−4.1, −60.6)	21.7	–43.0	–42.8	–0.2 ± 0.6
		BA-like	–79.4 (−22.4, −57.0)	32.4	–47.0	–25.4	**–21.6** **±** **1.7**

The MD simulations performed for Po revealed similar structural stability for the two binding modes, especially in the second half of the trajectories (Figures 7 and S6). Thus, the RMSD profiles for the protein backbone were in the range 1.5–2.0 Å, whereas values around 2.0 Å were obtained for the binding cavity, and about 1.5 Å (CA-like mode) and between 1.2 and 2.5 Å (BA-like mode) for the ligand. Nevertheless, the analysis of the RMSF plots revealed the occurrence of larger fluctuations when Po adopted the CA-like binding pose, mainly affecting residues 225–275, 350–370 and helix 12 (Figure S7). In turn, a more compact structure (SASA = 13841 ± 19 Å^2^; Table S3) was obtained the BA-like complex compared to Po in the CA-like one (SASA = 14800 ± 15 Å^2^; Table S3). In the case of Hede, with the only exception of a transient structural change affecting helix H12 (at about 400 ns), the RMSD profiles were stable along the second half of the trajectory for the CA-like mode (average RMSD of 1.9 and 1.4 Å for the binding pocket and the ligand, respectively (Figures 7 and S8). In contrast, a larger rearrangement of the ligand occurred in the BA-like binding mode, although a stable binding mode was reached along the last 300 ns of the MD simulation (Figures 7 and S8). Larger fluctuations in the C-terminus of helix 11 and helix 12 were observed for the CA-like complex (Figure S8), leading to a more compact structure (SASA of 13799 ± 15 and 14324 ± 18 Å^2^ for the CA-like and CA-like complexes; Table S3).

The MM-GBSA calculations supported the BA-like binding mode of both Po and Hede. Whereas the van der Waals component was roughly similar for all the complexes, the preference for the BA-like binding mode could be attributed to the more favorable electrostatic interaction (Table 2). Indeed, the per-residue decomposition of the interaction energy revealed distinct patterns of electrostatic interactions for CA and BA in their native complexes (Figures 8; S10 and S11), and the preservation of these electrostatic contacts for Po and Hede upon binding in the BA-like arrangement, which was stabilized by the interaction with Lys367 and favorable contacts with Arg288, Arg280, Lys261, Lys263 and Lys265, while there was a partial destabilization with Glu365 and Asp362, and Glu343.

**Figure 8:**
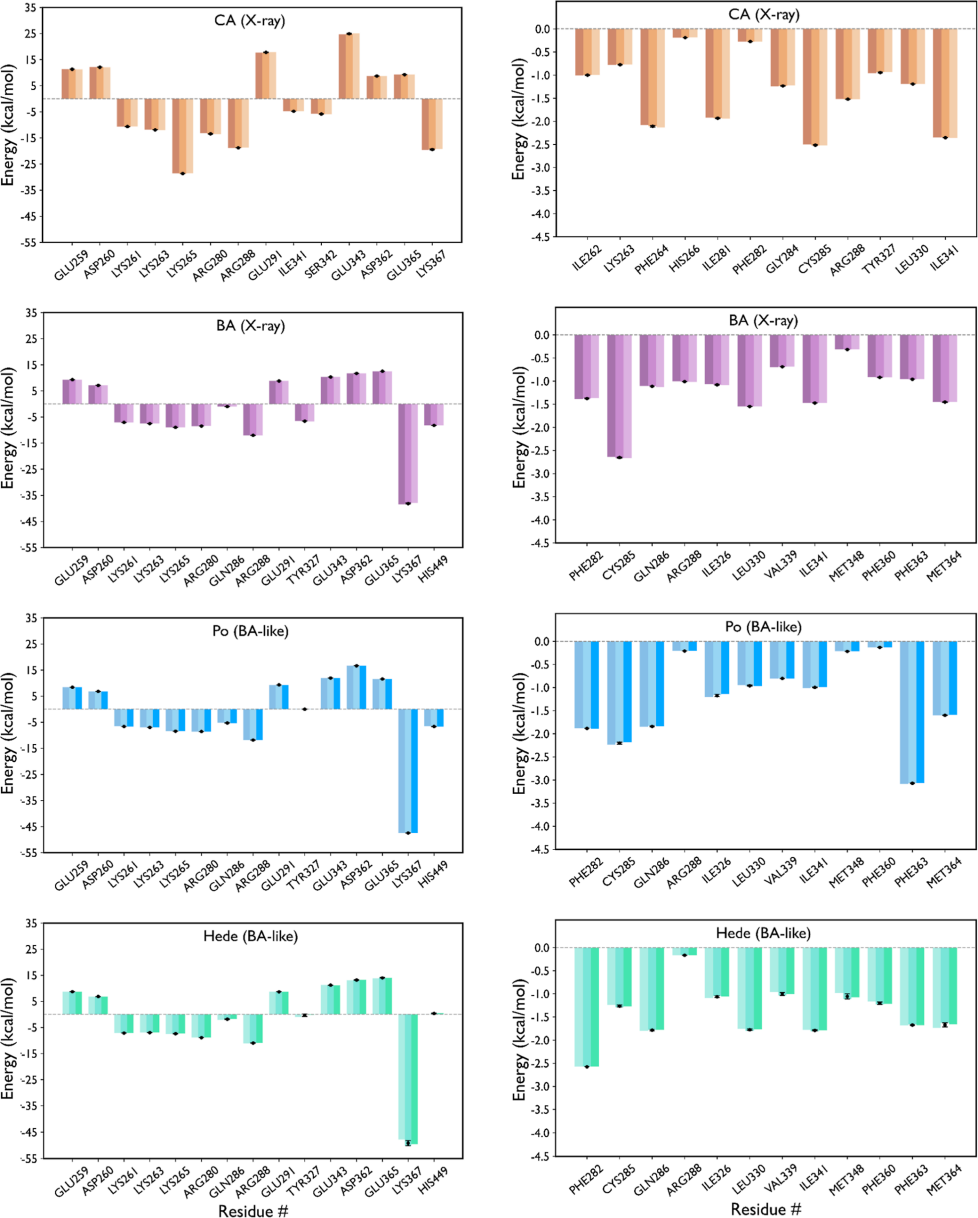
Per-residue energetic decomposition of the electrostatic and van der Waals interaction energies. Values determined for (top) CA in the native X-ray complex, and (from middle to bottom) BA, Po and Hede in the BA-like arrangements. Electrostatic and van der Waals components are given in the left and right columns. All values in kcal/mol. Average ±SD values among replicas are denoted with black dots and lines, respectively.

It is worth noting that upon binding of Po and Hede in the BA-like orientation, the structural distortion observed in helices H11 and H12 in the X-ray structure of the complex with BA reported by Brusotti et al.^23^ is maintained along the MD simulations (Figure 9). Thus, the inclusion of these ligands leads to conformational changes in both H11 and H12 helices due to the distinct arrangement of Po and Hede in the binding pocket, which mimics the spatial pose of BA and differs from the pose adopted by rosiglitazone (Figure 9). The structural distortions caused by the ligand shift are expected to alter the conformational flexibility of the receptor and in turn affect the coactivator recruitment. Accordingly, this distortion could represent a key structural determinant that supports the antagonist behavior observed for these ligands.

**Figure 9:**
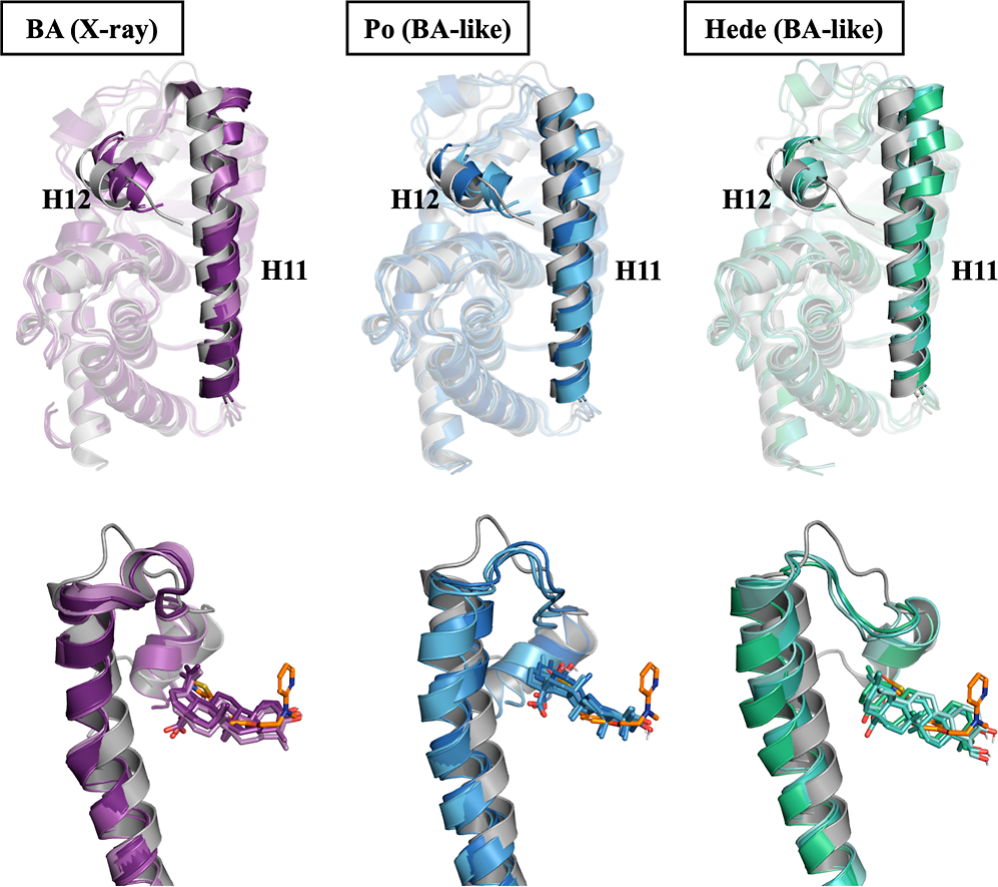
Structural rearrangement of helices H11 and H12 after ligand binding to the LBD in PPARγ. The structural distortion of helices H11 and H12 observed in the X-ray structure of the complex with BA is preserved along the trajectories sampled for the complexes with BA (left), Po (center) and Hede (right). (Top) superposition of the final snapshots collected from the three independent MD simulations run for complexes with BA (purple), Po (blue) and Hede (green) and the rosiglitazone-PPARγ crystallographic structure (PDB entry: 2PRG; gray). (Bottom) detail of rosiglitazone (orange sticks), BA (purple sticks), Po (blue sticks) and Hede (green sticks) and the position of H11 and H12 helices (for the sake of clarity, other structural elements are not shown).

## Discussion

In this study, Pomolic acid (Po) and Hederagenin (Hede), two structurally related pentacyclic triterpenoids occurring in *R. canina*, are identified as ligands of PPARγ with antagonistic and selective modulatory properties. Our findings demonstrate that small structural differences between closely related triterpenoids can translate into markedly distinct functional and mechanistic outcomes at the level of PPARγ signaling. This supports the emerging concept that fine-tuned modulation of PPARγ, rather than full receptor activation, may underlie the health effects attributed to certain dietary bioactive compounds and provide metabolic benefits while avoiding the adverse effects associated with classical agonists.^5,81^

Both Po and Hede significantly reduced lipid accumulation during 3T3-L1 adipocyte differentiation and efficiently antagonized rosiglitazone-induced PPARγ transactivation, without displaying intrinsic agonist activity in reporter assays (Figures 4 and 5). These effects were consistently observed across cellular differentiation and transcriptional models and agree with previous studies describing pentacyclic triterpenoids such as betulinic acid, oleanolic acid derivatives, and protopanaxatriol, as PPARγ antagonists that limit adipogenesis and modulate lipid metabolism in preclinical models.^23,25,29,82^ Despite these shared phenotypic effects, Po and Hede important differences emerged at the transcriptional level.

Po strongly repressed the expression of key adipogenic and lipogenic genes, including *Glut4* and *Fasn*, particularly at higher concentrations (Figure 4C). This transcriptional repression is consistent with previous reports describing that Po and its derivatives inhibit adipocyte differentiation, but also fatty acid synthase, acetyl-CoA carboxylase and glycerol-3-phosphate dehydrogenase activities in 3T3-L1 cells, which could further contribute to its antiadipogenic effects.^46,47,83^

In contrast, Hede displayed a distinct transcriptional profile. Although it reduced intracellular triglyceride accumulation to an extent comparable to Po (Figure 4D,E), Hede consistently induced the expression of several canonical PPARγ target genes involved in lipid uptake and intracellular lipid handling, including *Fabp4*, *Plin1*, *Cd36*, and *Glut4* (Figure 4F).

In line with these observations, previous studies have shown that Hede enhances lipid metabolism and promotes adipose tissue browning, favoring a more oxidative and thermogenic phenotype over lipid storage.^48^ Consistently, transcriptional analysis revealed increased expression of genes involved in lipid mobilization and fatty-acid oxidation (*Atgl* and *Cpt1a*), in response to Hede treatment (Figure 4F). The induction of these genes suggests activation of pathways related to lipolysis and mitochondrial fatty-acid utilization, supporting enhanced lipid turnover rather than lipid storage, consistent with a metabolically favorable PPARγ modulation profile. This mechanism may explain the reduction in intracellular lipid accumulation observed despite the increased expression of several adipogenic markers. Collectively, these findings support that Hede may act as a food-derived selective modulator of PPARγ rather than a pure antagonist ligand.^28,48^

This dissociation between lipid storage and gene induction is a defining feature of selective PPARγ modulators, which uncouple adipogenic activity from metabolically favorable transcriptional programs.^6–9^ Such selective modulation of PPARγ has been associated with improved lipid handling, enhanced mitochondrial function, and increased metabolic flexibility, thereby contributing to the maintenance of lipid homeostasis, while avoiding the excessive adipogenic effects typically elicited by full PPARγ agonists.^11,15,71,84,85^ In this context, molecules such as diosmin and chelerythrine have been shown to interact with PPARγ with atypical binding modes or partial agonism, improving insulin sensitivity and promoting browning of white adipose tissue without inducing marked adipose tissue expansion or weight gain.^12,14^ Some of these ligands appear to modulate PPARγ phosphorylation status and coregulator recruitment, including the selective engagement of coactivators such as PGC-1α, thereby favoring fatty-acid oxidation, mitochondrial programs, and thermogenic gene expression over lipogenesis.^71,86–88^

Mechanistic insight into these differential effects was obtained from ligand-binding and coregulator recruitment assays. Both compounds bound directly to the PPARγ ligand-binding domain with micromolar affinity (Figure 6A,D) and antagonized rosiglitazone-induced receptor transactivation (Figure 5). However, neither Po nor Hede promoted recruitment of the NCoR-1 corepressor (Figure 6C,F), distinguishing them from inverse agonists that actively suppress basal receptor activity.^69^ Notably, Po failed to recruit the TRAP220 coactivator under all experimental conditions tested (Figure 6B), consistent with its silent/neutral antagonistic profile.^69,77^ By contrast, Hede, while antagonistic in transcriptional reporter assays, induced partial recruitment of the TRAP220 coactivator, reaching approximately 20% of the maximal response elicited by the full agonist GW1929 (Figure 6E). This partial efficacy suggests that Hede stabilizes an intermediate PPARγ conformation that permits limited coactivator interaction without triggering full transcriptional activation, consistent with a selective PPARγ modulatory profile.

Importantly, these effects are strongly context dependent. In reporter assays based on artificial promoters containing only the PPAR response element (PPRE) and where the chromatin context is simpler and additional regulatory elements are absent, both compounds behave similarly as antagonists. In contrast, in adipocytes, endogenous genes are regulated within a more complex chromatin environment that includes multiple regulatory regions in each gene and involves the coordinated action of different transcription factors, coactivators, corepressors, and chromatin-remodeling complexes. Under these conditions, ligand-specific receptor conformations can differentially modulate coregulator recruitment, resulting in gene-specific transcriptional responses (Figure 10).

**Figure 10:**
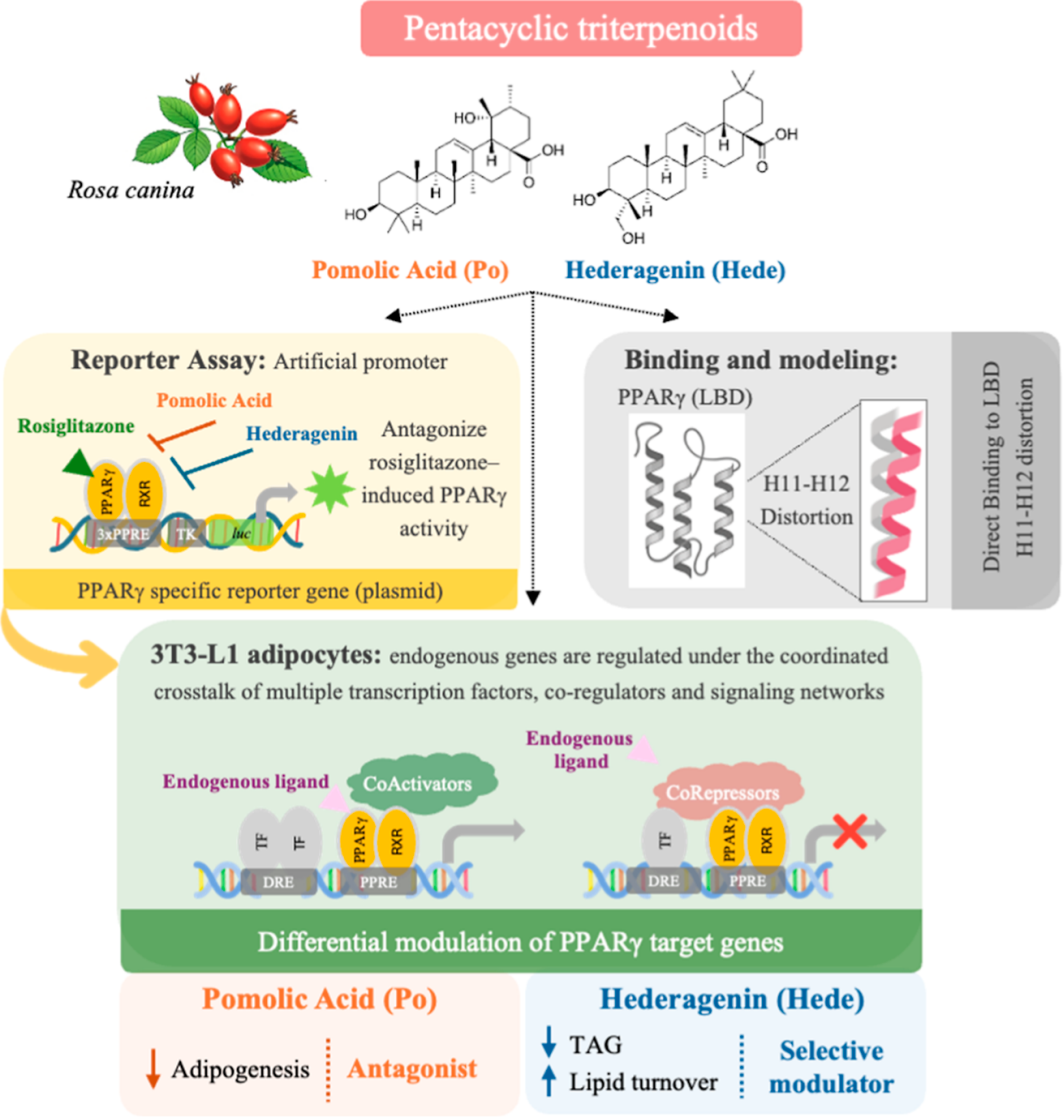
Context-dependent modulation of PPARγ by Pomolic acid (Po) and Hederagenin (Hede). Both compounds bind to the PPARγ ligand-binding domain and induce conformational changes affecting helices H11–H12. In luciferase reporter assays performed in HepG2 cells where transcription is driven by an artificial promoter, both compounds act as antagonists of PPARγ. In contrast, in 3T3-L1 adipocytes, where endogenous genes are regulated within a chromatin environment involving, several regulatory elements per gen, multiple coregulators and transcription factors, Po behaves as a neutral antagonist, whereas Hede acts as a selective modulator, leading to differential metabolic gene regulation.

Consistent with our findings, low-potency partial agonism of Hede (EC_50_ ≈ 37.8 μM) has been reported in alternative cellular contexts,^89^ supporting the notion that its transcriptional output may vary depending on the cellular environment. In agreement with this context-dependent behavior, PPARγ modulation is well documented and reflects the dynamic nature of receptor conformational ensembles as well as tissue-specific coregulator availability. For example, BADGE acts as an PPARγ antagonist in NIH-3T3 cells but functions as a partial agonist in ECV304 cells,^73,74,90^ whereas CDDO-Me displays antagonistic activity in CV-1 cells,^26^ yet behaves as a partial agonist in SW-480 cells.^75^ Notably, in the latter system, CDDO-Me promoted recruitment of multiple coactivators (SRCs, TRAP220, PGC1α, and CARM) together with the corepressor SMRT in GAL4-based two-hybrid assays.^75^ Together, these observations underscore that PPARγ activity reflects a dynamic equilibrium of ligand-stabilized receptor conformations and coregulator availability rather than a strict binary agonist–antagonist classification.

It is also worth noting that the transcriptional outcomes elicited by Po and Hede did not follow a strictly monotonic concentration–response relationship. Several genes showed increased expression at intermediate concentrations but decreased expression at higher doses (Figure 4). Such nonmonotonic patterns are characteristic of hormetic dose–response relationships frequently reported for plant-derived bioactive compounds, in which low concentrations activate adaptive or compensatory cellular pathways whereas higher concentrations engage additional regulatory mechanisms that result in different transcriptional outcomes.^91,92^ Similar biphasic responses have been described for dietary phytochemicals such as genistein and resveratrol.^93–97^ This behavior further supports the idea that ligand-specific stabilization of distinct receptor conformations can generate diverse transcriptional outcomes depending on cellular context.

Molecular modeling and MD simulations further provided the structural basis underlying these functional differences. Both Po and Hede adopted a BA—like binding orientation within the PPARγ LBD, positioning the carboxylate group toward helices H11 and H12. This binding mode was energetically favored over the CA-like orientation (Table 2), mainly due to more favorable electrostatic interactions, particularly with residues Lys367, Arg288, Arg280, Lys261, and Lys265 (Figure 8). Importantly, our simulations pointed out that binding of Po and Hede preserved the structural distortion of helices H11 and H12 observed in the BA-PPARγ X-ray complex (Figure 9). Because these helices are critical for the formation of the AF-2 coactivator binding surface, their altered conformation impair coactivator docking, thus providing a basis to explain the lack (Po) or partial (Hede) TRAP220 recruitment observed experimentally. This provides a structural rationale for the antagonistic behavior of Po and the weak partial agonism of Hede.

Complementary molecular dynamics analyses further supported the stability of the ligand–receptor complexes. Root-mean-square fluctuation (RMSF) and solvent-accessible surface area (SASA) analyses indicated subtle differences in receptor flexibility and binding pocket exposure depending on the bound ligand, providing additional insight into the dynamic behavior of the complexes and reinforcing the concept that Po and Hede stabilize distinct conformational states of the receptor.

Notably, Hede exhibited slightly lower receptor flexibility and subtle differences in electrostatic interactions, which may facilitate partial coactivator engagement and selective transcriptional activation, in line with current models of SPPARγM action.^69,98,99^

In this regard, structural features of the ligands may also contribute to their functional differences. Classical thiazolidinedione agonists such as rosiglitazone possess relatively flexible structures that facilitate adaptation within the Y-shaped ligand-binding pocket of PPARγ and stabilization of the fully active receptor conformation. In contrast, the rigid pentacyclic scaffold characteristic of triterpenoids such as Po and Hede may restrict conformational adaptability within the binding pocket, favoring alternative receptor conformations associated with partial agonism or selective modulation.

Importantly, the seemingly minor differences in the chemical structure of Po and Hede are sufficient to trigger distinct activity profiles upon interaction with PPARγ. In turn, this highlights the subtle balance of the interactions that may be formed from specific chemical groups attached to the pentacyclic triterpenoid skeleton in regulating the overall conformational plasticity of the receptor, and their impact on the assembly with other molecular partners. In this context, the flexible structure of full agonists, such as rosiglitazone, may facilitate the adoption of the bioactive conformation required for efficient PPARγ-mediated transcriptional activation.

These findings align with structural and biophysical studies demonstrating that ligand-specific stabilization of PPARγ conformational ensembles governs coactivator engagement and transcriptional output.^67,70,77,100,101^ Within this framework, Po appears to preferentially stabilize a transcriptionally inactive receptor conformation, whereas Hede allows limited formation of the AF-2 coactivator-binding surface, supporting selective coactivator recruitment and gene activation without full adipogenic commitment.

From a food chemistry and nutritional perspective, these findings establish a molecular framework linking *R. canina* triterpenoids to the modulation of lipid metabolism through differential modulation of PPARγ. Po emerges as a food-derived silent PPARγ antagonist with strong antiadipogenic activity, whereas Hede acts as a selective PPARγ modulator that preserves metabolically relevant gene expression while limiting lipid accumulation. The coexistence of these distinct modulatory profiles within *R. canina* offers a mechanistic explanation for the antiobesity and metabolic benefits previously reported.^32,41,43,44^

Subtle structural differences among these closely related pentacyclic triterpenoids translate into distinct receptor modulatory profiles and cellular outcomes, reinforcing selective PPARγ modulation as a nutritionally relevant strategy to improve lipid handling without full receptor activation and its associated adverse effects.

From a translational perspective, selective PPARγ modulation represents a promising avenue for metabolic disease management. These natural triterpenoids combine antiadipogenic, lipid-modulating, and anti-inflammatory properties while circumventing the liabilities of synthetic full agonists, and their capacity to restrict adipocyte expansion while promoting lipid mobilization and oxidative programs positions them as valuable molecular scaffolds for next-generation PPARγ-targeted interventions in obesity and type 2 diabetes. Moreover, given emerging evidence that PPARγ phosphorylation contributes to insulin sensitization independently of adipogenesis,^71,87^ the selective profile of Hede raises the possibility that this regulatory axis may also be involved, warranting further investigation.

## Supplementary Material



## Notes

Table S1. Oligonucleotide sequences for RT-qPCR analysis. Table S2. Coregulator peptides and their sequences used in binding assays. Figure S1. Gene induction during 3T3L1 differentiation in nontreated cells. Figure S2. Root-Mean Square Deviation (RMSD; Å) profiles for simulations performed for the complex with caulophyllogenin (CA). Figure S3. Root-Mean Square Fluctuation (RMSF; Å) profiles for simulations performed for the complex with caulophyllogenin (CA) Figure S4. Root-Mean Square Deviation (RMSD; Å) profiles for simulations performed for the complex with betulinic acid (BA). Figure S5. Root-Mean Square Fluctuation (RMSF; Å) profiles for simulations performed for the complex with betulinic acid (BA) Figure S6. Root-Mean Square Deviation (RMSD; Å) profiles for simulations performed for the complex with pomolic acid (Po). Figure S7. Root-Mean Square Fluctuation (RMSF; Å) profiles for simulations performed for the complex with pomolic acid (Po) Figure S8. Root-Mean Square Deviation (RMSD; Å) profiles for simulations performed for the complex with hederagenin (Hede). Figure S9. Root-Mean Square Fluctuation (RMSF; Å) profiles for simulations performed for the complex with hederagenin (Hede) Figure S10. Per-residue energy decomposition (kcal/mol) of the van der Waals contribution for (left) CA-like and (right) BA-like arrangements of the ligand. Figure S11. Per-residue energy decomposition (kcal/mol) of the electrostatic contribution for (left) CA-like and (right) BA-like arrangements of the ligand. (PDF)

## Abbreviations

ACS: American Chemical Society
ATGL: adipose triglyceride lipase
BA: betulinic acid
CA: caulophyllogenin
CD36: platelet glycoprotein 4-fatty acid translocase
CPT1a: carnitine palmitoyltransferase 1a
DMEM: Dulbecco’s Modified Eagle’s Medium
DOAJ: Directory of Open Access Journals
FASN: fatty acid synthase
FABP4 (aP2): fatty acid binding protein 4
GLUT4: glucose transporter 4
Hede: hederagenin
LD: linear dichroism
MD: molecular dynamics
MM-GBSA: molecular mechanics–generalized born surface area
PLIN1: perilipin 1
Po: pomolic acid
PPARγ: peroxisome proliferator-activated receptor gamma
ACACA: acetyl-CoA carboxylase alpha
TAG: triglyceride
Rosi: rosiglitazone.
