# Supplementary material for: Distinctive Behavior and Selective Modulation of PPARγ by Pentacyclic Triterpenoid Pomolic Acid and Hederagenin from Rosa canina
Source: J Agric Food Chem. 2026 Apr 30;74(18):14376–92. doi: 10.1021/acs.jafc.5c17657 (PMC13178073; doi:10.1021/acs.jafc.5c17657)

SUPPORTING INFORMATION

Distinctive Behavior and Selective Modulation of PPARγ by Pentacyclic Triterpenoid Pomolic Acid and Hederagenin from *Rosa canina*

Mariano Nicola-Llorente[1,2‡], Francisco J. Hermoso-Pinilla[1,3‡], Daniel Torres-Oteros[1,2], F. Javier Luque[1,3,4], Silvia Canudas[1,2], Pedro F. Marrero[1,3,5], Diego Haro[1,3,5], Joana Relat[1,2,5*].

[1]*Department of Nutrition, Food Sciences and Gastronomy, School of Pharmacy and Food Sciences, Food Torribera Campus, University of Barcelona, Santa Coloma de Gramenet, 08921, Spain.*

[2]*Institute of Nutrition and Food Safety of the University of Barcelona, INSA-UB Maria de Maeztu Unit of Excellence, Santa Coloma de Gramenet, 08921, Spain.*

[3]*Institute of Biomedicine of the University of Barcelona (IBUB), Barcelona, 08028, Spain.*

[4]*Institute of Theoretical and Computational Chemistry (IQTCUB). Barcelona, 08028, Spain.*

[5]*Centro de Investigación Biomédica en Red de Fisiopatología de la Obesidad y Nutrición (CIBEROBN), Instituto de Salud Carlos III, Madrid, 28029, Spain.*

Table S1. Oligonucleotide sequences for RT-qPCR analysis.

Gene		Sequence	Code
B-Actin	Beta-Actin	F-5'-GCTCTGGCTCCTAGCACCAT<br>R-5'-GCCACCGATCCACACAGAGT	NM_007393.5
B2m	Beta-2 Microglobulin	F-5'-ACTGATACATACGCCTGCAGAGTT<br>R-5'-TCACATGTCTCGATCCCAGTAGA	NM_009735.3
M36b4	Ribosomal protein, large, P0 (Rplp0)	F-5'-AGATGCAGCAGATCCGCAT<br>R-5'-GTTCTTGCCCATCAGCACC	NM_007475.5
acaca	Acetyl-Coenzyme A carboxylase alpha	F-5'-TGTACAAGCAGTGTGGGCTGGCT<br>R-5'-CCACATGGCCTGGCTTGGAGGG	NM_133360.2
cd36	Platelet glycoprotein 4, fatty acid translocase	F-5'-ATGACGTGGCAAAGAACAGCT<br>R-5'-AAGGCTCAAAGATGGCTCC	NM_001421120.1
fabp4	Fatty acid binding protein 4	F-5'-ATTTCCTTCAAACTGGGCGT<br>R-5'-GGTCGACTTTCCATCCCACT	NM_001409513.1
fasn	Fatty acid synthase	F-5'-GCTGCGGAAACTTCAGGAAAT<br>R-5'-AGAGACGTGTCACTCCTGGACTT	NM_007988.3
glut4	Solute carrier family 2 (facilitated glucose transporter), member 4	F-5'-ACTCATTCTTGGACGGTTCCTC<br>R-5'-CACCCCGAAGATGAGTGGG	NM_001359114.1
plin1	Perilipin 1	F-5'-GATGCCCTGAAGGGTGTTAC<br>R-5'-CCTCTGCTGAAGGGTTATCG	NM_175640.2
pparγ	Peroxisome proliferator activated receptor gamma	F-5'-GCATCAGGCTTCCACTATGGA<br>R-5'-AATCGGATGGTTCTTCGGAAA	NM_001127330.2
atgl	Adipose Triglyceride lipase	F-5'-CGCCTCTCGAAGGCTCTCT<br>R-5'-TGTAGCCCTGTTTGCACATCTC	NM_001163689.1
cpt1a	Carnitine palmitoyltransferase 1a	F-5'-AGAATCTCATTGGCCACCAG<br>R-5'-CAGGGTCTCACTCTCCTTGC	NM_013495.2

Table S2. Coregulator peptides and their sequences used in binding assays.

**Coregulator peptide**	**Sequence**
TRAP220/DRIP-2	NTKNHPMLMNLLKDNPAQD
NCoR1 ID2	DPASNLGLEDIIRKALMGSFDDK

Table S3. Solvent-Accessible Surface Area (SASA; Å^2) of PPARg-ligand complexes. The SASA was computed considering the last 200 ns of the three independent simulations performed for the complexes with caulophyllogenin (CA), betulinic acid (BA), hederagenin (Hede) and pomolic acid (Po) adopting the CA-like (PDB entry: 5F9B) and BA-like (PDB entry: 5LSG) orientations.

	**CA-like**			
**Compound**	**R1**	**R2**	**R3**	**Average ± SD**
**CA**	14612	14626	14630	**14623 ± 10**
**BA**	14099	14106	14147	14117 ± 26
**Po**	14817	14792	14790	14800 ± 15
**Hede**	14314	14314	14345	14324 ± 18

	**BA-like**			
**Compound**	**R1**	**R2**	**R3**	**Average ± SD**
**CA**	14743	14770	14788	14767 ± 23
**BA**	13717	13698	13683	**13700 ± 17**
**Po**	13825	13836	13862	**13841 ± 19**
**Hede**	13783	13802	13812	**13799 ± 15**

Figure S1. To provide appropriate context for the transcriptional effects observed upon treatment, Fig. S1 includes a reference gene expression profile during 3T3-L1 adipocyte differentiation to illustrate the strong induction of adipogenic and lipid metabolism–related genes in untreated cells. The magnitude of this induction (in some cases >50–100-fold vs. undifferentiated cells) was not incorporated into the main figures (Fig. 4C and 4F), as it would compress the scale and obscure the effects of pomolic acid and hederagenin, limiting comparison between treatments. These data are therefore provided separately to support interpretation while preserving figure clarity.

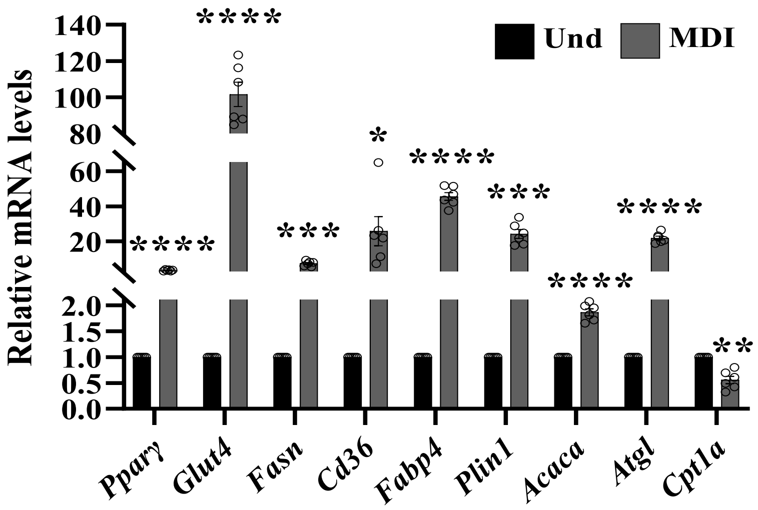

Figure S1. Gene induction during 3T3L1 differentiation in non-treated cells. 3T3-L1 preadipocytes were differentiated using the MDI cocktail and gene expression of adipogenic markers was analyzed by RT-qPCR (n = 6). Data are presented as mean ± SEM. Statistical significance: *p<0.05, **p<0.01, ***p<0.001, ****p<0.0001. Und = undifferentiated cells; MDI = differentiated cells.

Figures S2-S5. To validate the Molecular Dynamics simulations, the structural stability and binding behavior of the reference ligands caulophyllogenin (CA) and betulinic acid (BA) were analyzed in both CA-like and BA-like orientations. RMSD and RMSF profiles are provided to assess protein and ligand stability, as well as residue flexibility, supporting the consistency of the native binding modes observed in the crystallographic structures.

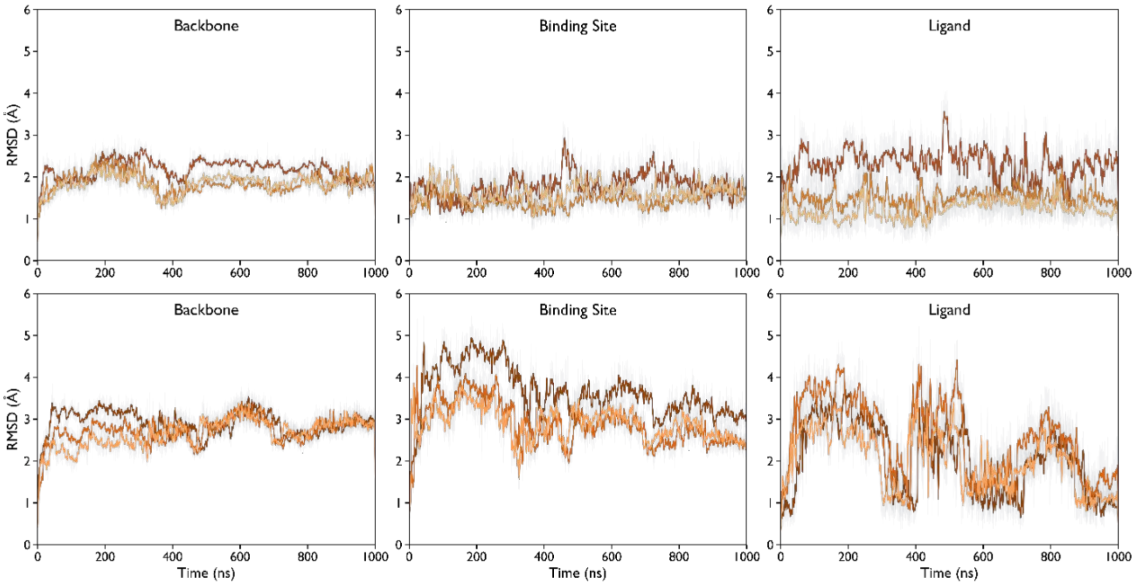

Figure S2. Root-Mean Square Deviation (RMSD; Å) profiles for simulations performed for the PPARg complex with caulophyllogenin (CA), which was arranged in (top) the CA-like and (bottom) the BA-like orientations. The RMSD was determined for (left) the protein backbone, (middle) binding site, which was defined as the set of residues within 4Å of the ligand, and (right) ligand. Each triplicate is represented using a distinct color.

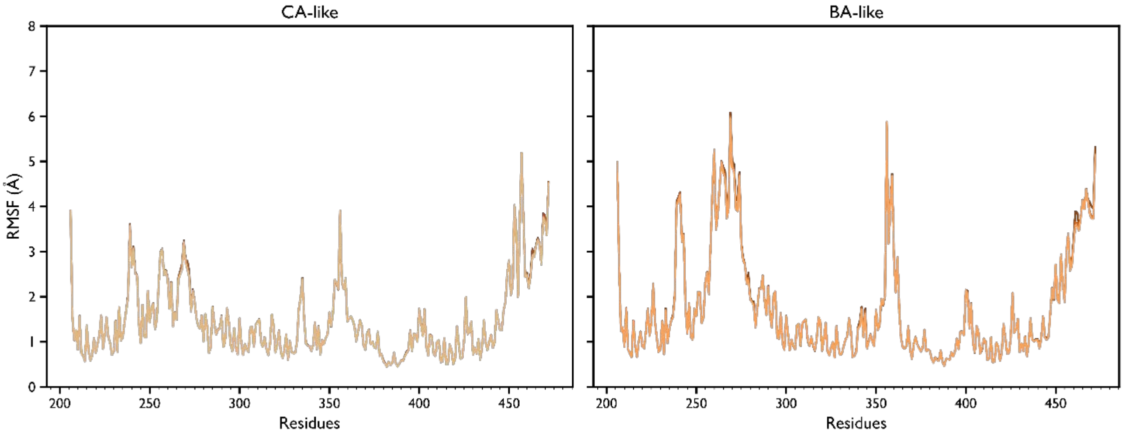

Figure S3. Root-Mean Square Fluctuation (RMSF; Å) profiles for simulations performed for the complex with caulophyllogenin (CA) arranged in the (left) CA-like and (right) BA-like pose. Each triplicate is represented using a distinct color.

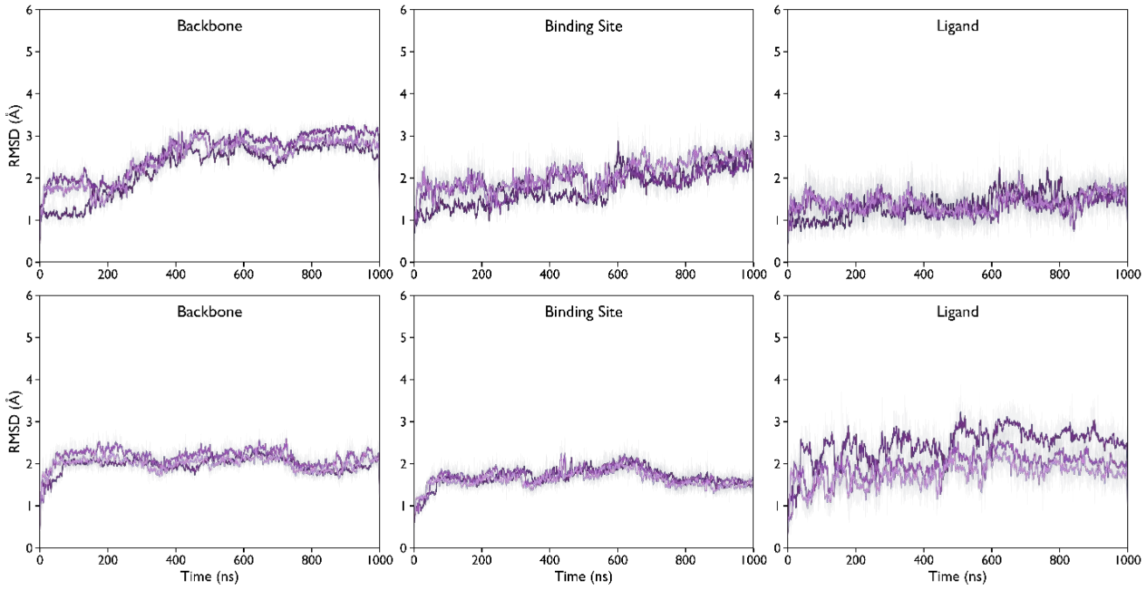

Figure S4. Root-Mean Square Deviation (RMSD; Å) profiles for simulations performed for the complex with betulinic acid (BA), which was arranged in (top) the CA-like and (bottom) the BA-like orientations. The RMSD was determined for (left) the protein backbone, (middle) binding site, which was defined as the set of residues within 4Å of the ligand, and (right) ligand. Each triplicate is represented using a distinct color.

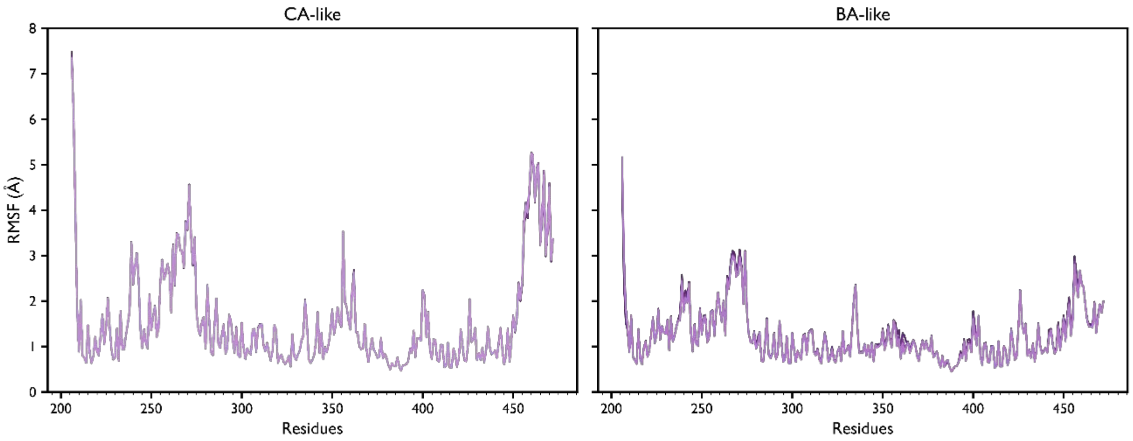

Figure S5. Root-Mean Square Fluctuation (RMSF; Å) profiles for simulations performed for the complex with betulinic acid (BA) arranged in the (left) CA-like and (right) BA-like pose. Each triplicate is represented using a distinct color.

Figures S6-S9. MD simulations were performed to characterize the binding mode of pomolic acid and hederagenin bound to the PPARγ ligand-binding domain. RMSD and RMSF plots are shown to evaluate the stability of the protein-ligand complex, and receptor flexibility, providing insight into ligand-dependent conformational changes, particularly in regions relevant for coregulator interaction.

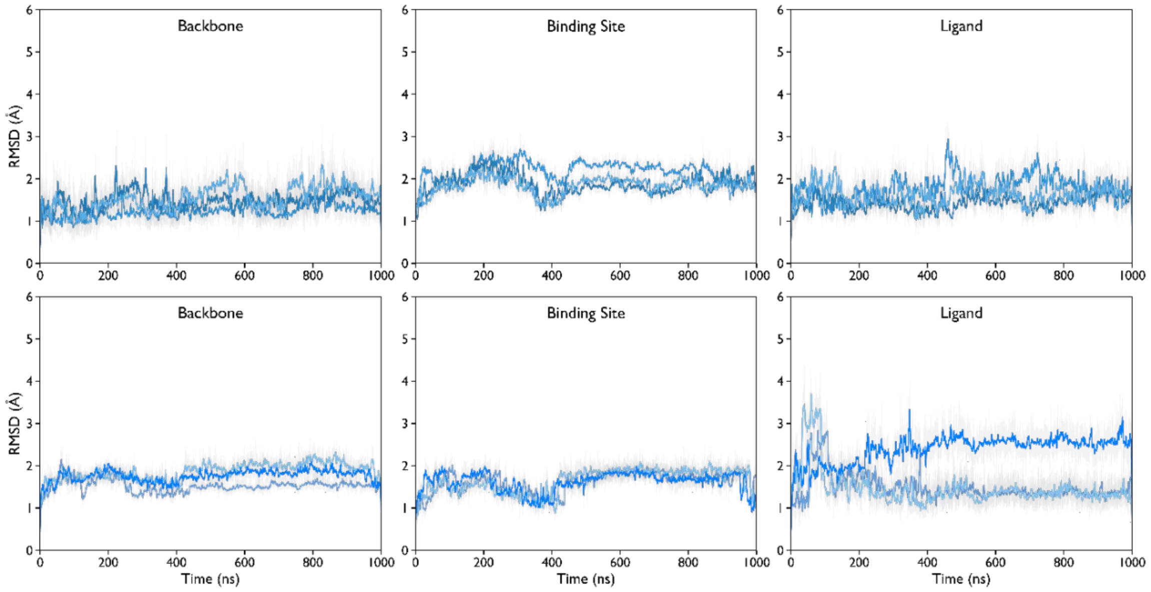

Figure S6. Root-Mean Square Deviation (RMSD; Å) profiles for simulations performed for the complex with pomolic acid (Po), which was arranged in (top) the CA-like and (bottom) the BA-like orientations. The RMSD was determined for (left) the protein backbone, (middle) binding site, which was defined as the set of residues within 4Å of the ligand, and (right) ligand. Each triplicate is represented using a distinct color.

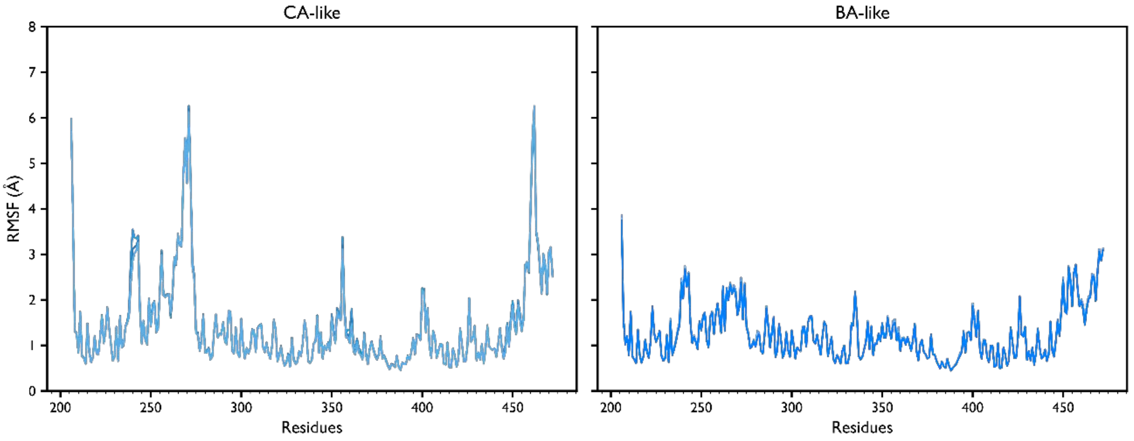

Figure S7. Root-Mean Square Fluctuation (RMSF; Å) profiles for simulations performed for the complex with pomolic acid (Po) arranged in the (left) CA-like and (right) BA-like pose. Each triplicate is represented using a distinct color.

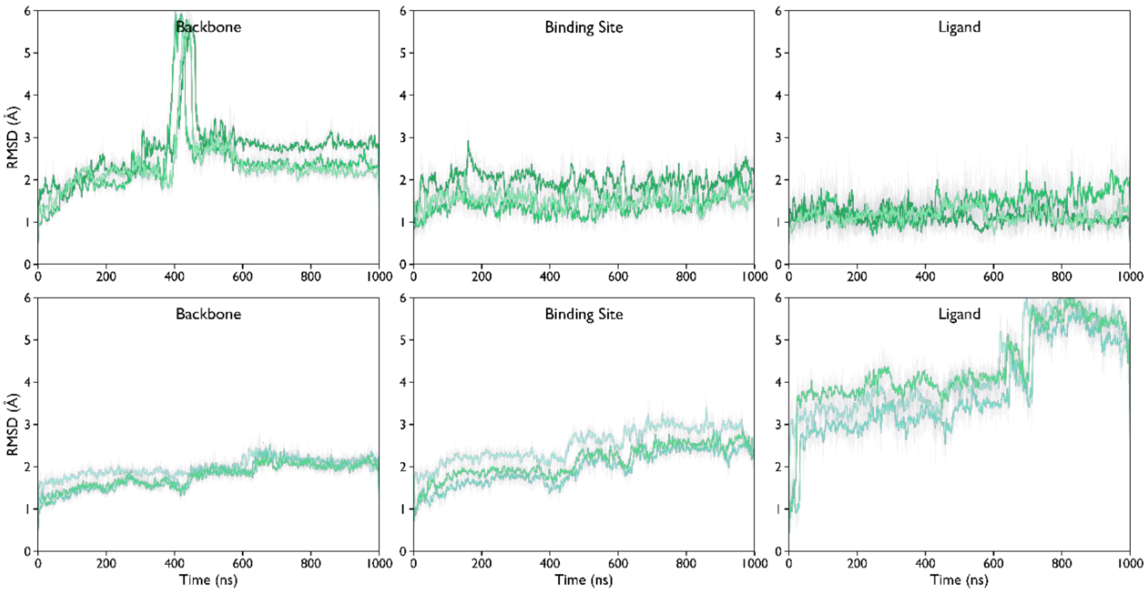

Figure S8. Root-Mean Square Deviation (RMSD; Å) profiles for simulations performed for the complex with hederagenin acid (Hede), which was arranged in (top) the CA-like and (bottom) the BA-like orientations. The RMSD was determined for (left) the protein backbone, (middle) binding site, which was defined as the set of residues within 4Å of the ligand, and (right) ligand. Each triplicate is represented using a distinct color.

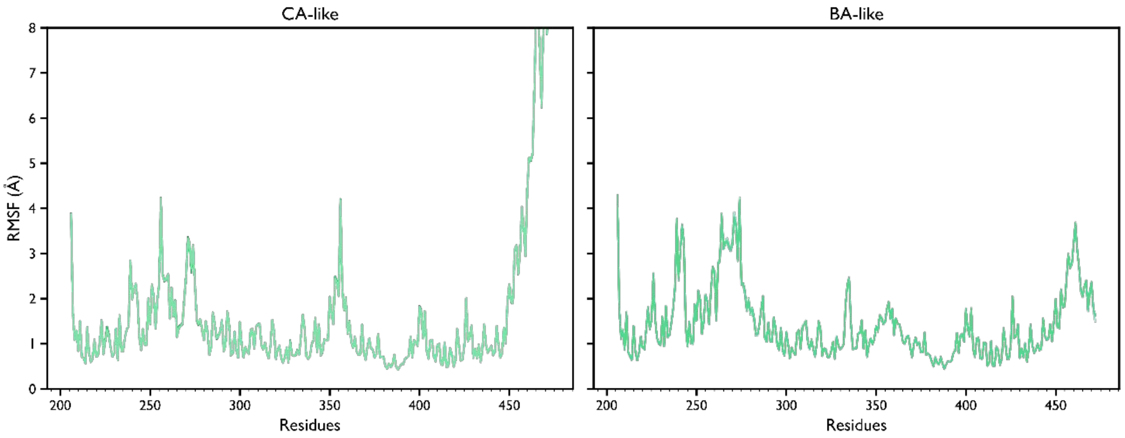

Figure S9. Root-Mean Square Fluctuation (RMSF; Å) profiles for simulations performed for the complex with hederagenin (Hede) arranged in the (left) CA-like and (right) BA-like pose. Each triplicate is represented using a distinct color.

Figure S10. Per-residue energy decomposition (kcal/mol) of the van der Waals contribution for (left) CA-like and (right) BA-like arrangements of the ligand. This analysis aimed to identify key residues contributing to the ligand binding considering the last 200ns of the simulation. Residues with an interaction energy (in absolute value) lower than 0.5 kcal/mol were excluded for the sake of clarity. The average value is shown as a black dot, and the standard deviation is indicated by error bars. Each replica of the triplicate is shown using a distinct color.

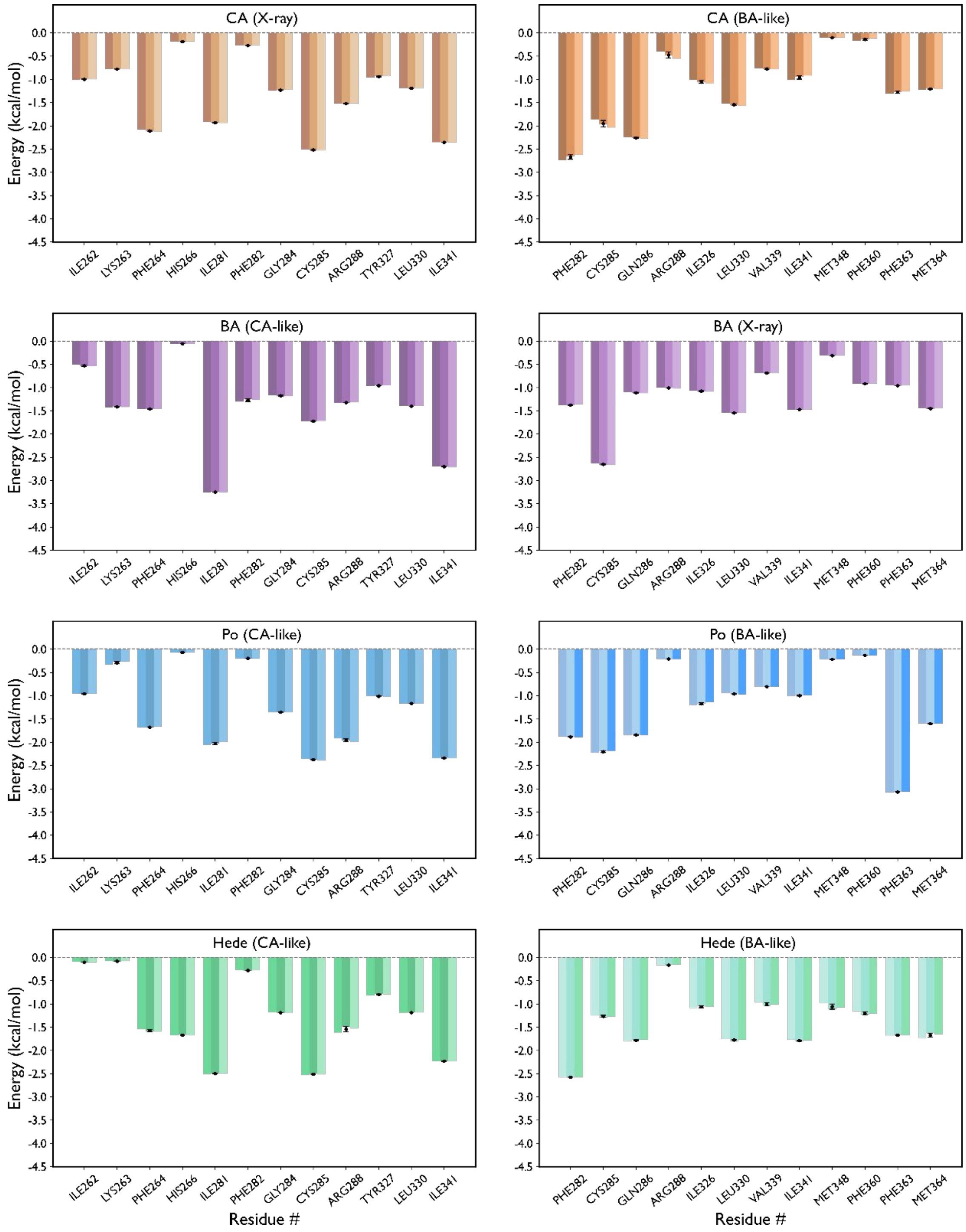

Figure S11. Per-residue energy decomposition (kcal/mol) of the electrostatic contribution for (left) CA-like and (right) BA-like arrangements of the ligand. This analysis aimed to identify key residues contributing to the ligand binding considering the last 200ns of the simulation. Residues with an interaction energy (in absolute value) lower than 5.0 kcal/mol were excluded for the sake of clarity. The average value is shown as a black dot, and the standard deviation is indicated by error bars. Each replica of the triplicate is shown using a distinct color.

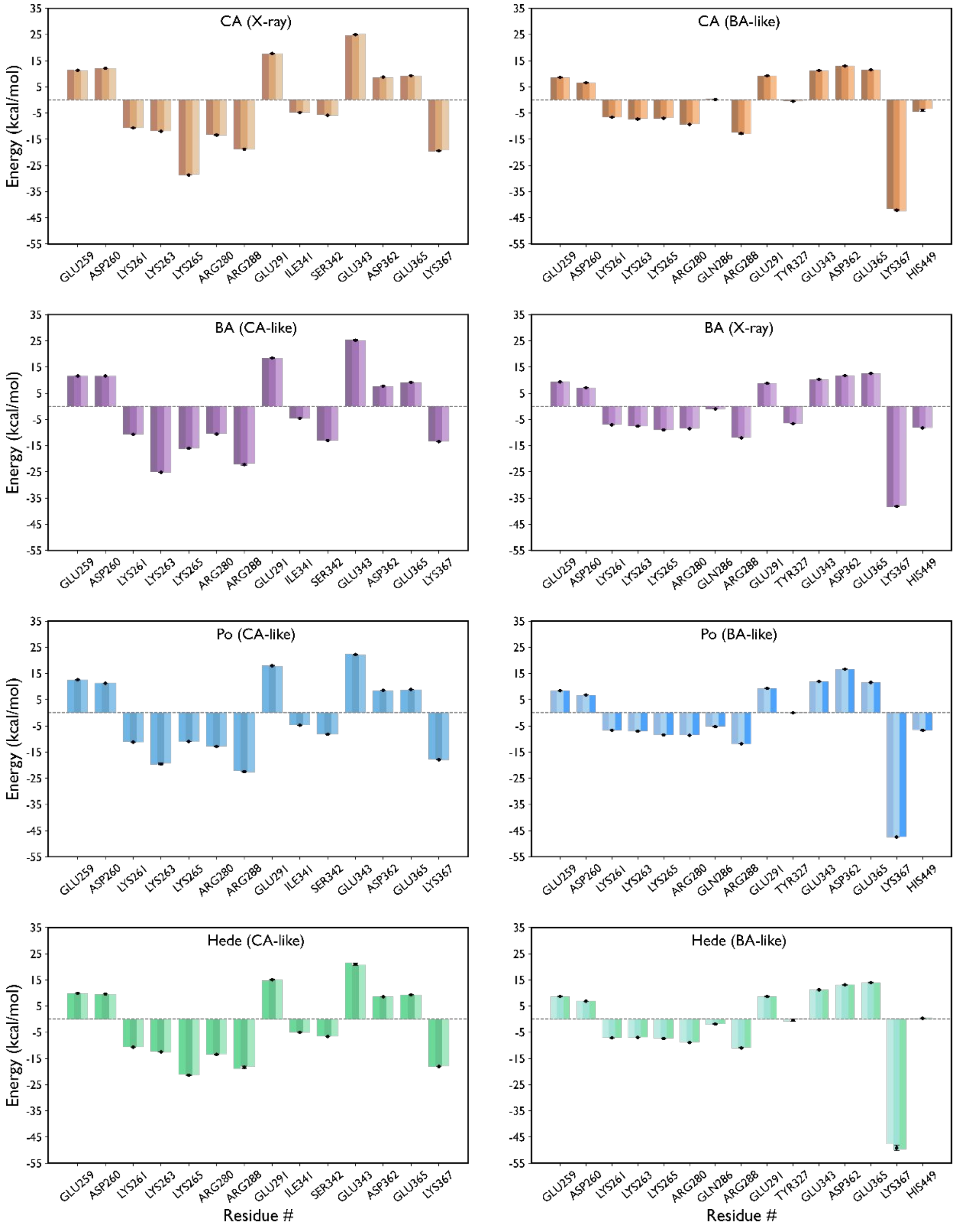

Supplement: Supplementary file 1 [file jf5c17657_si_001.pdf]
